# A Microfluidics-Based Ultrahigh-Throughput Screening Unveils Diverse Ketoreductases Relevant to Pharmaceutical Synthesis

**DOI:** 10.1021/acs.analchem.5c01029

**Published:** 2025-09-16

**Authors:** Laura Blas-Muñoz, Alejandro H. Orrego, Michael Hofmeister, John Martínez-Salvador, Carmen Ortega, Vanessa Rondón Berrio, Jorge Díaz-Rullo, James Finnigan, Simon Charnock, Wolf-Dieter Fessner, José Eduardo González-Pastor, Aurelio Hidalgo

**Affiliations:** § Centro de Biología Molecular Severo Ochoa, Universidad Autónoma de Madrid-Consejo Superior de Investigaciones Científicas (UAM-CSIC), Nicolás Cabrera 1, Madrid 28049, Spain; ∥ Department of Molecular Biology, Universidad Autónoma de Madrid, Campus de Cantoblanco, Madrid 28049, Spain; ⊥ 26536 Technische Universität Darmstadt, Institute of Organic Chemistry and Biochemistry, Alarich-Weiss-Str. 4, Darmstadt 64287, Germany; # Centro de Astrobiología, (CSIC-INTA), Ctra de Torrejón a Ajalvir, km 4, Torrejón de Ardoz 28850, Spain; ∇ Prozomix Ltd., Building 4, West End Ind. Estate, Haltwhistle, Northumberland NE49 9HA, United Kingdom; ○ Instituto de Biología Molecular Universidad Autónoma de Madrid, Nicolás Cabrera 1, Madrid 28049, Spain

## Abstract

Ketoreductases (KREDs) have become increasingly valuable biocatalysts due to their ability to produce chiral alcohols with high enantioselectivity. Prior to our work, Thai et al. developed an efficient and easy assay for their discovery, but the throughput was limited. Based on their work, we developed an ultrahigh-throughput screening assay to discover KREDs. First, we optimized Thai’s assay by adapting it to a droplet format and increased its throughput by combining droplet microfluidics and fluorescence-activated cell sorting (FACS). Then, we demonstrated that our new assay was reliable and sensitive by successfully screening a library of 1.5 million clones. This allowed us to discover KREDs with low identity with known enzymes or with a previously undescribed substrate scope, which could not have been predicted computationally. In conclusion, our assay was used to carry out the first metagenomic screening for KREDs in microdroplets, and it can be used to screen any large KRED library toward enzyme discovery or evolution, as well as to enable coupled ultrahigh-throughput screening assays for other enzyme activities.

## Graphical abstract

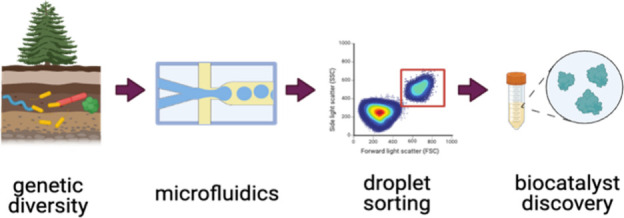

## Introduction

Enzymes are invaluable catalysts for numerous industrial applications, from pharmaceuticals to biofuels,^1^ meeting the principles of green chemistry and promoting a circular, biobased economy.^2,3^ The wide applicability of enzymes in biocatalysis, bioremediation, microbial fermentations, and sustainable agriculture calls for the creation of methods for their rapid discovery and optimization. Traditional methods for enzyme discovery have relied heavily on the cultivation of independent microbes, often limiting the ability to harvest enzymes from unculturable microorganisms. On the contrary, metagenomics accesses the genetic material from environmental samples (metagenomes) directly, bypassing the need for cultivation.^4,5^ Metagenomics has transformed the field of enzyme discovery, leading to identify novel enzymes with unparalleled functionalities, exceptional thermostability, solvent tolerance, and specificity for various substrates, with tremendous potential in industrial processes, environmental remediation, and biotechnological applications.^6–11^

Metagenomic screening can be either sequence- or activity-driven. Activity-based metagenomic screening involves creating large clone libraries from environmental DNA and interrogating them for the desired function. Unlike sequence-based metagenomics, the naïve, activity-based screening has the advantage of finding innovative biological solutions that are unbiased by a query search sequence.^12^ However, it demands assays that are able to specifically measure the desired activity with sufficient sensitivity and throughput. The long and tedious activity-based mining of metagenomes for novel enzymes has been revolutionized by microfluidics, through the use of microscopic water-in-oil (w/o) droplets as independent reaction compartments, resulting in unprecedented throughput and a reduction of costs, and use of reagents.^13–15^ Some microfluidic operations (e.g., droplet making) require using specialized equipment, while others (e.g., droplet sorting) can be carried out using equipment available in many research facilities, such as fluorescence-activated cell sorters (FACS).^16^ This contributes to the democratization of ultrahigh-throughput screening.

Ketoreductases (KREDs, EC 1.1.1.x) are oxidoreductases that reversibly catalyze the reduction of ketones to secondary alcohols using cofactors, such as reduced nicotinamide adenine dinucleotide (NADH) or nicotinamide adenine dinucleotide phosphate (NADPH), to facilitate the transfer of electrons. They have become increasingly useful in industrial and pharmaceutical applications due to their ability to produce chiral alcohols with high enantioselectivity, which is particularly useful for synthesizing enantiopure pharmaceuticals.^17–20^ Previous to our work, Thai et al.^21^ developed a fluorescence-based assay in microtiter plate format for the discovery of KREDs useful in the synthesis of chiral carbinols, recurring building blocks in many therapeutic agents.^22^ They assayed KRED activity via the conversion of an alcohol into a fluorescent ketone. However, regardless of its simplicity, the format of a microtiter plate and the reaction volume limit the throughput of the screening campaigns for enzyme discovery and engineering. Based on their work, we developed a new ultrahigh-throughput screening to discover KREDs using microfluidics and FACS. We then demonstrated its reliability and sensitivity by screening a large metagenomic library for new KREDs (Figure 1). This way, we discovered several enzymes that are active against diverse secondary alcohols, including some building blocks relevant for pharma and fine chemistry. Moreover, we uncovered a putative substrate scope for glucose dehydrogenase (GDH), a well-known class of ketoreductases, which could not have been predicted computationally.

**Figure 1:**
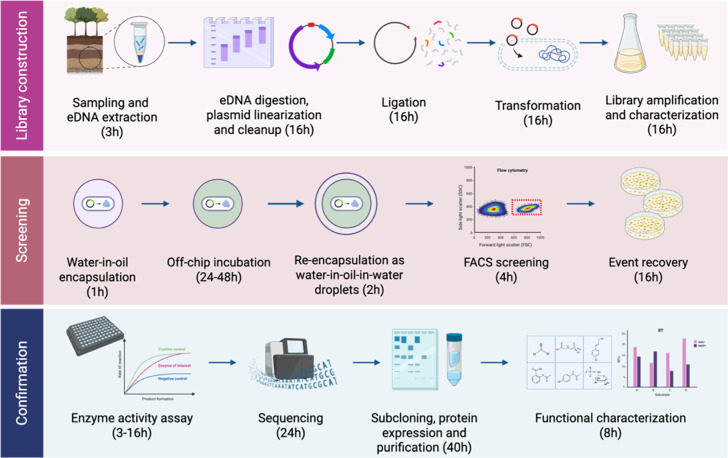
Workflow for metagenomic library construction, screening using droplet microfluidics, and confirmation of hits. Library construction: environmental DNA was extracted from soil, digested, ligated into a linearized pBluescript II SK (+) plasmid, and transformed in *E. coli* DH10B to create a library of 1.5 million individuals and 1.1 kbp average insert size. Library screening: an aliquot of the library was encapsulated in water-in-oil (w/o) droplets together with alcohol **3**, incubated for 48 h, and re-encapsulated as water-in-oil-in-water droplets that were sorted by FACS to recover the KRED-positive droplets by plating on LB Amp. Hit confirmation: clones were grown, induced, confirmed for KRED activity, sequenced and the candidate open reading frame was identified, subcloned, expressed, purified, and characterized against a panel of secondary alcohols. Image created with Biorender.com.

## Materials and Methods

### Materials

Specific materials used in this work are reported in Table S1 (oligonucleotides), Table S2 (plasmids), Table S3 (*Escherichia coli* strains), and Table S4 (culture media) and were used according to the manufacturer’s instructions. Materials and procedures used for microfluidic device microfabrication and emulsification are included in the Supplementary Methods. The design for the flow focusing device design is reported in Figure S1 and was kindly provided by the Hollfelder group. Materials and procedures for organic synthesis are described in the Supporting Information.

### Assay Development

To test the leaking of the fluorescent ketones **2** and **4**, a 1 mM solution of each ketone in 2% (v/v) dimethyl sulfoxide (DMSO) in the ZY autoinduction medium was encapsulated in water-in-oil (w/o) droplets that were afterward mixed with w/o droplets containing 2% (v/v) DMSO in ZY medium. The resulting emulsions were mixed in a 1:1 ratio (v/v); photographs were taken every 24 h, using an Olympus BX50 fluorescence microscope with an FITC filter set, ×25 objective, and a Pike F032B Camera (Allied Vision) set at 15 ms of exposure. For a quantitative analysis of leaking, the fluorescence of at least 625 droplets was quantitated using the software Fiji.^23^

To establish the sensitivity of the method, we encapsulated several concentrations of ketone **4** in w/o droplets, re-emulsified them as w/o/w droplets, imaged the emulsion by brightfield and fluorescence microscopy to verify the integrity and quality of the emulsion, and analyzed it by FACS. Droplets were diluted 10:300 (v/v) in PBS-Tween80 and separated in a FACSAria Fusion flow cytometer (BD Biosciences, DiVA 8 software) using a 100 μm nozzle, a laser for excitation at 405 nm, and a filter of 525/50 nm for emission. Typical analysis was run at 20 psi, resulting in a throughput of 250 events/s. Data were processed using Floreada.io.

To find examples of inactive and active KREDs against the fluorogenic alcohol **3**, Prozomix KRED panels 1–4 were screened with this substrate. Panels containing 1 mg of freeze-dried enzyme per well were incubated with 20 μL of 1 mM alcohol **3** at room temperature for 6 h. Images were acquired using an Amersham IM680 imager. Then, plasmids pET28b-K293 and pET28b-K349 were electroporated into *E. coli* BL21­(DE3). Transformants were resuspended in ZY with 1 mM alcohol **3** and 2% v/v DMSO, encapsulated in w/o droplets with an average occupancy of 0.1 cell/droplet, and incubated in an orbital shaker for 48 h at 30 °C and 100 rpm. The excess oil was removed, and the emulsion was re-encapsulated as a w/o/w emulsion and immediately subjected to FACS analysis as described above. Events exceeding a predetermined fluorescence threshold were collected in 1.5 mL Eppendorf tubes with 200 μL of LB, incubated for 2 h at 37 °C, diluted, spread on LB Kan plates in triplicate, and cultivated at 37 °C. The identity of positive colonies was confirmed by polymerase chain reaction (PCR) using primers K349_Fw and K349_Rv.

### Screening of a Metagenomic Library

A metagenomic library was constructed with a sample of garden soil from the Centre of Astrobiology (CSIC-INTA) (40°30′12″ N, 3°27′54′′ W, authorization number ESNC107, ABSCH-IRCC-ES-258964-1) according to the procedure detailed in the Supporting Information. To screen the metagenomic library, clones were resuspended in fresh ZY supplemented with 1 mM alcohol **3** and 2% DMSO, encapsulated as single cells in w/o droplets, and incubated for 48 h at 30 °C and 100 rpm in an orbital shaker. The w/o emulsion was then re-encapsulated and immediately analyzed by FACS, and events considered positive were collected in 1.5 mL tubes with LB medium. FACS-positive droplets were plated directly on LB Amp and cultivated at 37 °C. Colonies were pooled, resuspended in LB Amp, and subjected to screening for two additional consecutive rounds.

### Hit Recovery and Characterization

To determine the size of the recovered metagenomic fragments, plasmids from the grown colonies were purified and digested with XbaI and *Hin*dIII. To confirm the KRED activity of the metagenomic hits, 169 random colonies from the 3 rounds were grown in a 96-well plate with ZY Amp for 16 h at 30 °C and 180 rpm. Cells were harvested and washed twice with PBS 1X, and the pellet was resuspended in 1.5 U/μL of benzonase and 1 mg/mL lysozyme. Plates were incubated for 30 min at 37 °C, subjected to freeze–thaw cycles and centrifuged at 3600 × g and 4 °C. Then, 100 μL of supernatant was mixed with a reaction mix of 1 mM alcohol **3**, 2% DMSO (v/v) in 50 mM phosphate buffer pH 7.5, and either NAD^+^ or NADP^+^, monitoring the increase in fluorescence (Exc. 340 nm; Em. 520). To rule out false positives due to phenotypic variability, *E. coli* DH5α was transformed with the isolated plasmids from the colonies in which KRED activity had been detected. After that, they were assayed again for KRED activity, as described above. Clones with the highest activity were sequenced using the T3 and T7 primers, and the putative ORFs were detected as described above. The putative ORFs were then used for searches using BlastP^24^ and InterPro.^39^ Where no KRED functional assignment was obtained, structures were predicted with AlphaFold^40^ and used as search queries in Foldseek.^41^

The putative KRED-encoding ORF sequences were ordered and subcloned into pET28b­(+) for recombinant expression in *E. coli* BL21­(DE3) using ZY Kan, at 25 or 30 °C for 16 h or at 20 °C for 24 h. A pellet of 1 mL of each induced culture was analyzed by SDS-PAGE to determine the localization of the produced protein.^23^ If there was no discernible protein expression, the pET28b construct was cotransformed with the Takara plasmid pKJE7, transformants were selected on LB Kan and chloramphenicol, and protein expression was attempted again. If the protein of interest was in the soluble fraction, cultures were centrifuged at 4000 × g for 30 min and resuspended in 30 mL of Tris–HCl 50 mM pH 7.5 buffer, lysed using a pressure homogenizer, and centrifuged at 15,000 × g for 30 min. Proteins were purified using ion-metal affinity chromatography (Ni-NTA Superflow, Qiagen) according to the manufacturer’s instructions. The purified protein preparations were dialyzed against Tris–HCl 50 mM NaCl 100 mM pH 7.5 (Spectra/Por 6–8 kDa, Spectrum laboratories) at 4 °C and stored at 4 °C until assayed. Protein concentration was quantified using the Bio-Rad Protein Assay (Bio-Rad) with bovine serum albumin (BSA) as a standard.^29^

### Functional Characterization of KREDs against a Panel of Diverse Alcohols

A reaction mixture was assembled consisting of 0.1 mg/mL of pure protein, 1 mM of each alcohol, and 5 mM NAD^+^ or NADP^+^ in PBS with 2% (v/v) DMSO. The reaction was initiated by the addition of enzyme, and the reduction of the cofactor was determined by monitoring the increase of absorbance at 340 nm in a Fluostar Optima plate reader (BMG Labtech). The activity was calculated using a calibration curve of each reduced cofactor ranging from 0 to 2 mM. A blank reaction with no enzyme was used to determine background activity and subtract it from the measurements. All measurements were carried out in triplicate.

A sequence similarity network (SSN) was generated using the Enzyme Similarity Tool (EFI-EST) using the FASTA sequences of all expressed ORFs as input and completing up to ca. 10,000 sequences with randomly chosen members of families related to ketoreductases (SDR, MDR, and AKR) such as PFAM00106, 00107, 13561, 08240, and 00248.^29–31^ Nodes represent sequences with 100% identity, and the identity cutoff for edges was 40%. The SSN was visualized using Cytoscape v3.10.2.

## Results and Discussion

### Assay Development

We sought to adapt the KRED assay of Thai et al.^21^ to a droplet format, so we first examined the leakiness of ketone **2**, from w/o droplets. Upon mixing 1:1 (v/v) w/o droplets containing ketone **2** with droplets containing only medium, we observed that the fluorescent ketone **2** was transferred from the droplets containing it within 1 min (Figure S2). To overcome this limitation, we introduced a charged dimethylpiperazinium moiety, yielding ketone **4** and the corresponding alcohol **3** (Scheme 1 and Figures S3–S6). This is congruent with previous research, in which the rate of interdroplet transport of the substrate has been shown to decrease with increasing the hydrophilicity of the fluorophore.^32^ We also verified that the absorption and emission wavelengths of ketone **4** remained basically unchanged with respect to those of ketone **2** (Figure S7). Using ketone **4**, the fluorescence exchange between the two droplet populations was found tolerable (both populations were distinguishable) until up to 48 h, and more noticeable after 72 h (Figure 2). In comparison with other fluorophores often used in droplet assays, this rate of interdroplet transport was lower than that of resorufin,^27^ rhodamine, and coumarin,^27,33^ but higher than that of fluorescein and pyranine.^34^ Therefore, fluorescein and pyranine would allow longer incubation times and a higher sensitivity of the assay; however, ketone **4** is less bulky than pyranine, reducing the possibilities of assay bias.^34^ We also established a calibration curve of ketone **4** up to a 1 mM concentration and determined the limit of detection of the method to be 4.1 μM (Figure S8).

**Scheme 1:**
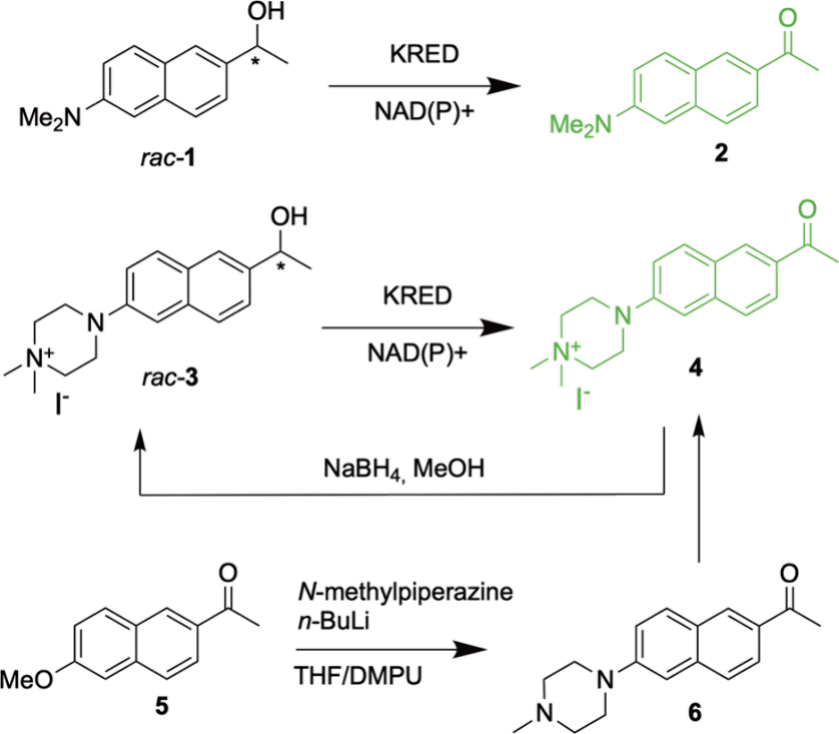
KRED Assay from Thai et al.^21^ (Top) and Synthesis of an Analogous, Droplet-Compatible Substrate (Bottom)

**Figure 2:**
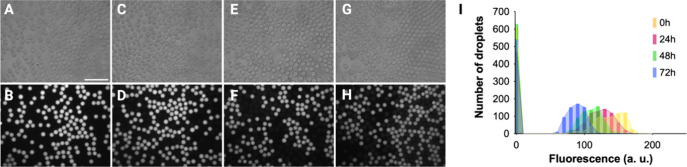
Leaking of fluorescent ketone **4** from droplets. Phase contrast (A,C,E,G) and fluorescence microscopy images at 100 ms of exposure (B,D,F,H) corresponded to a 1:1 mixture of droplets with and without ketone **4** after 0 (A,B), 24 (C,D), 48 (E,F), and 72 h (G,H). The fluorescence of >625 droplets per time point was quantitated using Fiji image processing software (I). Scale 100 μm.

Then, we screened panel numbers 1–4 of KREDs from Prozomix Ltd. (UK) to find two suitable enzymes to set up the methodology. KRED293 and KRED349 were chosen respectively as examples of inactive and active KREDs against alcohol **3** (Figure S9). Next, we encapsulated the corresponding *E. coli* transformants with KRED349-encoding plasmids as single cells in w/o droplets containing alcohol **3** and autoinduction medium and incubated the droplets for 48 h to verify the expected increase in activity with incubation time (Figure S10). The fluorescence increased from 24 to 48 h and so did fluorophore leaking, but it did not compromise the separation of the positive control. For this reason, we considered the 48 h time scale of incubation suitable to express and detect enzymes from metagenomic DNA fragments.^14^ Finally, to facilitate the recovery of sequence encoding hits after sorting, we opted to grow the single cells inside the droplets instead of using single-cell lysate assays. In this case, substrate and enzyme come together by partial and spontaneous cell lysis, supported by the presence of organic cosolvents in the droplets.^15^ We expected that growing the single cells in the droplets would produce more molecules of the fluorescent product, thus amplifying the assay signal.

Then, we prepared w/o emulsions harboring single *E. coli* transformants with KRED293- and KRED349-encoding plasmids and alcohol **3**, incubated them for 48 h then reinjected into a hydrophilic chip to obtain a water/oil/water (w/o/w) emulsion that could be analyzed by FACS (Figure 3 and Figure S11).

**Figure 3:**
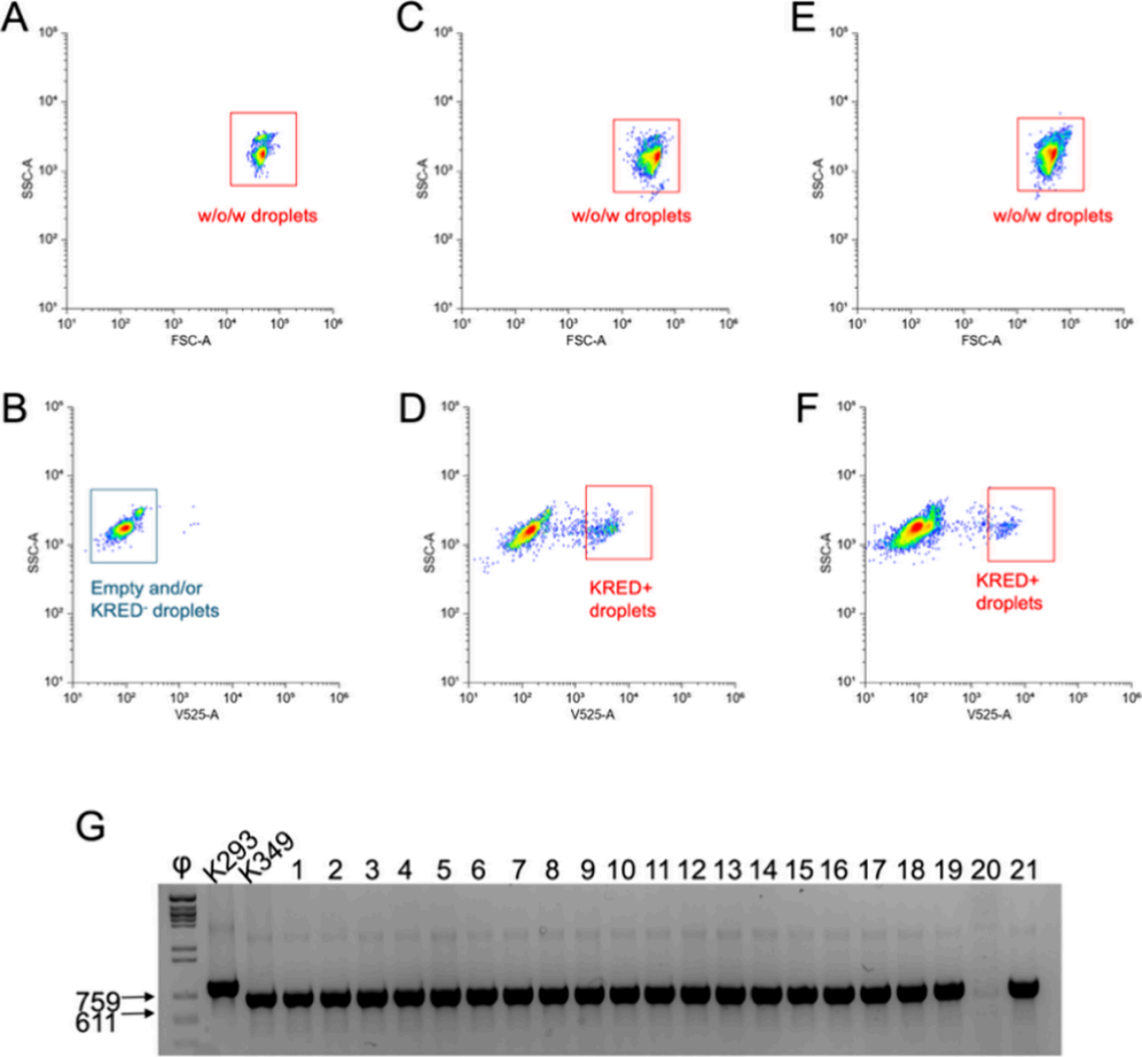
FACS analysis of water/oil/water (w/o/w) droplets encapsulating cells expressing the examples for KRED activity and confirmation by PCR. Dot-plots of side scatter (SSC) versus forward scatter (FSC) and of SSC versus the fluorescent signal at 525 nm of gated w/o/w droplets containing either cells expressing KRED293 (A,B), cells expressing KRED349 (C,D), or a mixture of the two populations in a ratio 5:1 (KRED293:KRED349) (E,F). The following number of droplets was analyzed for each sample: 8461 in panel (A), 6852 in panel (B), and 20,081 in panel (C). In all cases, there were also empty droplets intrinsically formed during the process of single-cell encapsulation. Colonies obtained from the sorted positive fraction of the 5:1 mixture were checked by colony PCR (G). The correct amplicon fragment confirming the presence of KRED349 was 611 bp. Φ DNA digested with Hind III was used as a fragment size marker.

Droplets with KRED293- and KRED349-expressing cells were analyzed either separately (Figure 3A,B and C,D, respectively) or combined in a 5:1 ratio (KRED293: KRED349, Figure 3E,F). In all cases, there was also a population of empty droplets as an intrinsic result of the strong dilution required for the non-deterministic encapsulation of single cells. As shown in Figure 3B, this population of empty droplets could not be distinguished from a population of droplets harboring cells that express an inactive KRED. We observed a wide separation between droplet populations containing KRED349-expressing cells and droplets containing either KRED293-expressing cells or empty cells (Figure 3B,D). Using the median fluorescence of KRED293- and KRED349-expressing cells in the droplets (112 and 2048, respectively), we established a 40:1 signal-to-noise ratio. Finally, we confirmed the presence of the KRED349-encoding plasmid in 21 random colonies grown from the sorted positive fraction of the 5:1 mixture (Figure 3G), reaching a purity of 95% and a calculated enrichment^16^ of 5.71-fold (of a maximum possible of 5.88-fold).

### Metagenomic Library Creation and Screening for Novel KREDs

We tested the assay by screening a metagenomic library of almost 1.5 million unique clones, harboring fragments of environmental DNA with an average insert size of approximately 1.1 kbp and containing an average of 1 open reading frame per fragment (Figure S12). Library clones were encapsulated as single cells in w/o droplets with alcohol **3**, incubated for 48 h, re-encapsulated, and analyzed by FACS as described above. Due to the difficulty to observe a clear positive population in the expected region of the plot, we aimed to sort approximately the top 1% droplets, plate them on a selective medium, pool the resulting colonies, and rescreen them two additional times (Figure S13). While the use of FACS instruments vs bespoke microfluidic sorters is one of the main advantages toward democratization of ultrahigh-throughput screening,^16,35,36,37^ their fixed excitation wavelengths may be disadvantageous for the sensitivity of the assay. For example, our instrument did not have a 375 nm laser (often used in FACS), which forced us to excite the droplets at a suboptimal wavelength of 405 nm. Using an optimal excitation wavelength would have increased the signal by 5-fold (Figure S14), enabling a reduction in the incubation time and, concomitantly, reducing the impact of leakage to improve the sorting accuracy.

The additional enrichment rounds increased the total screening time, but the use of viable cells within the droplets streamlined the recovery of the metagenomic clones, keeping the duration of each round under 1 week. Moreover, assays involving the growth of single cells from droplets may show less phenotypic variability compared to assays in which single cells are directly lysed and assayed upon encapsulation.^38^

### Hit Recovery and Characterization

We first confirmed that the size of the metagenomic fragments recovered from the grown positive cells was within the expected range (Figure S15) and, on average, longer than the input library, suggesting enrichment in fragments with a higher likelihood of harboring a complete ORF. We then randomly selected a minimum of 50 colonies from each round, incubated their corresponding induced lysates with alcohol **3** and NAD^+^ and NADP^+^ separately, and confirmed a significant KRED activity in 77 clones in total using a stringent arbitrary threshold of the average of the blank plus 5 times the standard deviation of the blank (Figure S16A,B). Then, to eliminate false positives due to phenotypic variability, we retransformed the plasmids from those 77 clones and verified whether the KRED activity was concomitantly transferred. We confirmed that 32 colonies in total displayed KRED activity (Figure S16C,D) and sequenced the 4 metagenomic clones with the highest KRED activity with NAD^+^ (3–1, 3–42, 3–45, 1–35) and NADP^+^(3–1, 3–42, 3–45, 2–7), for production and purification.

All of the metagenomic DNA fragments contained one or more plausible open reading frames (ORFs) (Figure S17). Therefore, to prioritize the ORFs with the highest likelihood of coding for KREDs, we used the ORF sequences as queries for searches in different databases. At first, we used a sequence-based approach (BlastP^24^ and Interpro^39^) and, if we did not obtain a clear prediction, we then used a structure-based approach by performing structure modeling through Alphafold^40^ and using the model as a query for a search in FoldSeek^41^ (Table S5). We found KRED candidate sequences in all sequenced metagenomic fragments, including a redundant sequence due to clonal amplification during the enrichment (e.g., metagenomic clones 3–42 and 3–45 were identical). We expressed the most likely codon-optimized candidate ORFs in *E. coli*. The ORFs 2–7–2, 3–1–1, and 3–42–1 were expressed successfully in the soluble fraction (Figure S18A), but ORF 1–35–2 could not be expressed at all, even in the presence of chaperones (Figure S18B), maybe because the sequence is too incomplete.

The purified proteins were assayed with a panel of diverse alcohols, some of which were precursors of active pharmaceutical ingredients or aroma compounds^42–44^ (Figure 4 and Figure S19). KRED 3–1–1 had ca. 79% sequence identity with glucose dehydrogenase (GDHs) (Table S5) and presented the highest activity and the widest substrate scope, including aromatic and aliphatic secondary alcohols. GDHs have been reported to accept nonsugar substrates, such as naphthoquinones, imines, and iminium salts, but neither phenylethanol nor short-chain alcohols.^45–47^ Moreover, KRED 3–1–1 showed a lax cofactor preference in the oxidation of alcohols **12** and **14**. Conversely, KRED 3–42–1 was specific for alcohol **12** and NADP^+^. Finally, the product of ORF 2–7–2 was inactive against all alcohols at room temperature (data not shown), which may be explained by multiple hypothetical reasons, among which are the difference between the screening and production host/vector systems, incompatibility with the N-terminal His-tag,^48^ the lack of specific chaperones,^49,50^ or inadequate assay temperature, considering that the closest BLAST hit is from a thermophilic microorganism (Table S5).

**Figure 4:**
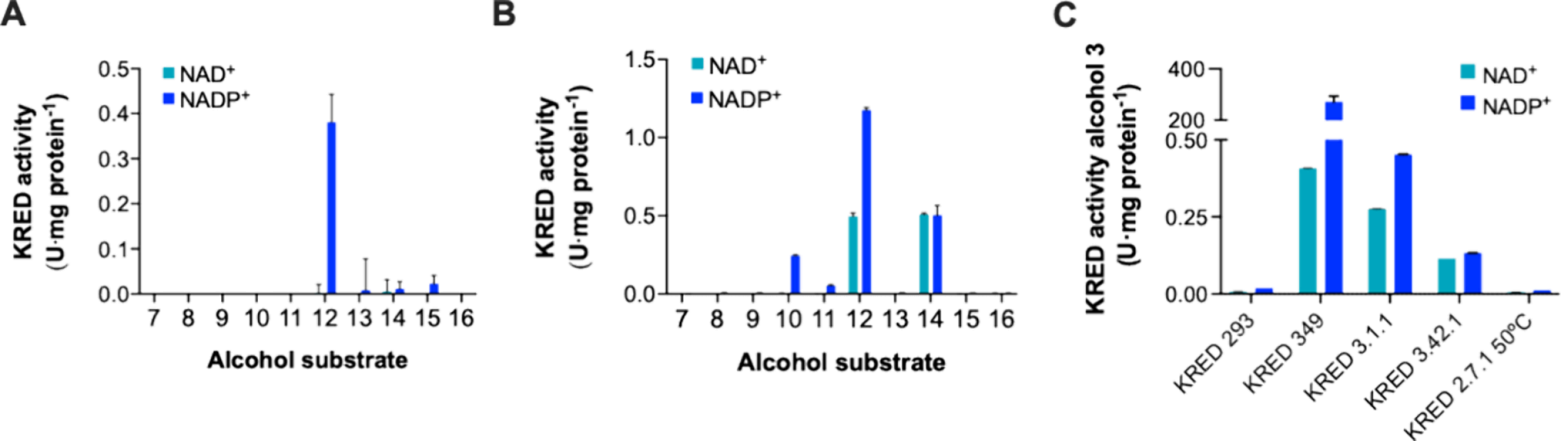
Activity of the soluble, purified proteins 3–42–1 (A) and 3–1–1 (B) against a panel of diverse alcohols, which can be found in Figure S19, as well as specific activity of control and discovered KREDs toward alcohol **3** (C). One activity unit corresponds to a micromole of reduced cofactor per min. Bars represent the average of *n* = 3 blank-corrected determinations, and error bars represent the standard deviation.

For KREDs 3–1–1 and 3–42–1, the largest activity was detected, with alcohols containing a 1-phenylethanol structural motif (alcohols 11, 12, and 14), partially similar to the structure of the screening substrate. Bias toward the screening substrate is a possible, well-known issue^51^ that would need to be more extensively confirmed and, if detected, it may be solved by the introduction of spacer arms between the functional group and the fluorophore,^52^ but it would complicate the synthesis of the fluorogenic substrate. Nevertheless, we were able to find KRED 3–1–1 that was active against one aliphatic alcohol, whose atypical substrate scope could not have been discovered bioinformatically. This discovery prompts the exploration of GDH and its close GDH homologues as biocatalysts beyond mere sugar oxidations and cofactor recycling.

In order to assess the found hits relative to the control KRED used to develop the assay, all purified proteins were assayed against alcohol **3** (Figure 4C). The large differences in specific activity suggested that the method was sensitive enough to detect weaker hits, with specific activity up to 2000-fold lower than that of the positive control KRED used to set up the assay (218 U vs 0.45 U mg protein^–1^). This was likely due to the 3 enrichment rounds as the most active hit, but also the weakest ones were found in round 3.

Lastly, we studied the uniqueness of KREDs from ORFs 2–7–2, 3–1–1, and 3–42–1 using an SSN (Figure S20). ORFs 3–1–1 and 3–42–1 clustered with other KREDs of the short-chain dehydrogenase/reductase (SDR) family, with ORF 3–1–1 connecting with many other SDRs and ORF 3–42–1 showing fewer connections. KRED 2–7–2 belongs to the Pfam group 00106, represented in the SSN, but did not cluster, as it has an identity lower than 40% with the represented sequences. This attests to the capacity of the assay to discover enzymes with low sequence identity to known KREDs or previously unassigned KREDs.

The proposed assay has several strengths. First, the substrate is directly converted into a fluorescent product with no need for coupled reactions, which have been reported before to assay KREDs.^53^ Second, separate enantiomers of alcohol **3** may be used to screen for KREDs with particular enantioselectivity, as illustrated previously.^21^ Third, the use of whole cells in droplets enabled the use of the cofactor pool present in the host, leading to the identification of KREDs irrespective of their cofactor preference; moreover, it made the assay more sensitive and the recovery of the hits easier and faster.^15^ Finally, the use of 2% (v/v) DMSO was a good compromise between substrate solubility, cell permeabilization, and cell viability and did not compromise droplet stability. This approach toward viable cell recovery is simpler than using a “tunable lysis” plasmid,^54^ which entails the need for transformation with an additional plasmid, and a previous titration of the lysis-inducing agent. We also recognize the weaknesses of our methodology, such as the background due to endogenous KREDs in the bacterial host, having to use a suboptimal excitation wavelength due to instrument constraints, and a leakage rate higher than those of common fluorophores, such as fluorescein. However, swift enrichment rounds allowed for the successful detection of KRED-containing metagenome fragments. Unfortunately, we detected fewer KREDs than Thai et al.^21^ using the racemic alcohol (approximately 10 vs 40% hits in Prozomix panel number 4). This difference is to be expected given the increased bulkiness and charged nature of *rac*-**3** compared with *rac*-**1**; however, it can be compensated by the increased throughput of our microfluidics-based screening. Moving forward with this methodology, increasing the hit rate and sorting accuracy is paramount. This could be achieved using complementary strategies. First, large-insert libraries (i.e., fosmid libraries) could be used as previously reported by Tauzin and co-workers,^15^ where metagenomic DNA inserts are larger and therefore coding sequences are less fragmented

Accelerating the identification of the responsible ORF in the metagenomic fragment will also be advantageous, e.g., by targeted knockout of suspect ORFs within the same vector and host in which they were screened, rather than subcloning. Also, the reduction of leakage and background can help increase the sorting accuracy. For the former, numerous strategies involving additives, increased pH or oil changes have been previously reported.^33^ For the latter, library expression could be carried out *in vitro*,^56–58^ abolishing the background reactions due to cytoplasmic dehydrogenases. Moreover, the *in vitro* expression would also decrease the duration of each screening round.^25^ Finally, since fluorogenic substrates do not exist for every enzyme of interest, a future application of our methodology could be in coupled enzyme assays. Reactions catalyzed by KREDs, such as the one described here, may be used in a more complex enzymatic cascade where the product of the main reaction cannot be easily determined by UV/vis or fluorescence spectrometry, e.g., for kinases,^26^ PETases,^28^ and many other enzymes.

## Conclusions

We developed an ultrahigh-throughput KRED screening assay based on a fluorogenic substrate whose synthesis is straightforward. We verified the reliability and sensitivity of our assay by screening a large metagenomic library using readily available FACS equipment and discovering novel KREDs, including enzymes relevant to pharmaceutical synthesis with different substrate scopes and cofactor preferences. To the best of our knowledge, this is the first published report of an enzyme discovery campaign for KREDs in ultrahigh-throughput. Moreover, our naïve strategy to interrogate the microbial diversity has provided insight into known enzymes and enzyme families. Therefore, our assay for ultrahigh-throughput screening of large libraries is an unmistakably promising solution to discovering, but also engineering, novel biocatalysts.

## Supplementary Material



## Notes

Microfluidic, microbiological, and organic chemistry methods; transfer of the fluorescent ketone; design of the flow focusing device; spectral properties of the original and modified fluorescent products of the KRED assay; images and cytometry plots supporting the calibration curve of ketone **4** in FACS; screening of the Prozomix KRED panels; images supporting the separation of control KREDs; graphic representation of ORFs in a sample of the library prior to screening; calibration curve of ketone **4** at different wavelengths; images and plots supporting library sorting; restriction analysis of plasmids from FACS-positive metagenomic library clones; KRED activity assays of randomly picked clones during each round; ORF prediction within the chosen metagenomic fragments; SDS-PAGE analysis of the expression of the discovered KREDs; panel of structurally diverse alcohols related to biocatalysis for characterizing the proteins with KRED activity; SSN of KRED families; NMR spectra of the ketones and secondary alcohols; oligonucleotide, plasmids, bacteria strains, culture media, and predicted ORFs included in the metagenomic hits (PDF)

## †

Heterogeneous Biocatalysis Group CIC biomaGUNE, Paseo de Miramón 182, Donostia 20009, Spain

## ‡

Department of Biological and Agricultural Engineering, North Carolina State University, 3110 Faucette Dr., Raleigh, North Carolina 27695–7625, USA
