# Supplementary material for: A Microfluidics-Based Ultrahigh-Throughput Screening Unveils Diverse Ketoreductases Relevant to Pharmaceutical Synthesis
Source: Anal Chem. 2025 Sep 16;97(38):20698–706. doi: 10.1021/acs.analchem.5c01029 (PMC12489886; doi:10.1021/acs.analchem.5c01029)
Supplement: Supplementary file 1 [file ac5c01029_si_001.pdf]

Supporting Information

A Microfluidics-Based Ultrahigh-Throughput Screening Unveils Diverse Ketoreductases Relevant to Pharmaceutical Synthesis

Laura Blas-Muñoz, Alejandro H. Orrego†, Michael Hofmeister, John Martínez-Salvador, Carmen Ortega, Vanessa Rondón Berrio‡, Jorge Díaz-Rullo, James Finnigan, Simon Charnock, Wolf-Dieter Fessner, José Eduardo González-Pastor, Aurelio Hidalgo*

Laura Blas-Muñoz - Email: laura.blas@uam.es, https://orcid.org/0000-0003-4213-1079; **Alejandro Herrera-Orrego** - Email: aherrera@cicbiomagune.es, https://orcid.org/0000-0003-4412-1579; **John Martínez-Salvador -** Email: john.martinez@uam.es, https://orcid.org/0009-0001-5513-7915; **Vanessa Rondón**-Email: vrondon@ncsu.edu, https://orcid.org/0000-0002-5932-5702. Centro de Biología Molecular Severo Ochoa Universidad Autónoma de Madrid-Consejo Superior de Investigaciones Científicas (UAM-CSIC), Nicolás Cabrera 1, Madrid 28049. Spain; Department of Molecular Biology, Universidad Autónoma de Madrid, Campus de Cantoblanco, Madrid 28049, Spain;

Michael Hofmeister- Email: michi.hofmeister@gmail.com; **Wolf-Dieter Fessner**- Email: wolf-dieter.fessner@tu-darmstadt.de, https://orcid.org/0000-0002-9787-0752. Technische Universität Darmstadt, Institute of Organic Chemistry and Biochemistry Alarich-Weiss-Str. 4, Darmstadt 64287, Germany,

Carmen Ortega - Centro de Biología Molecular Severo Ochoa Universidad Autónoma de Madrid-Consejo Superior de Investigaciones Científicas (UAM-CSIC), Nicolás Cabrera 1, Madrid 28049, Spain; Email: cortega@cbm.csic.es.

Jorge Díaz-Rullo - Email: jdiaz@cab.inta-csic.es, https://orcid.org/0000-0003-4545-9758; **José Eduardo González-Pastor**- Email: gonzalezpje@cab.inta-csic.es, https://orcid.org/0000-0002-7615-7042

Centro de Astrobiología, (CSIC-INTA), Ctra de Torrejón a Ajalvir, km 4, Torrejón de Ardoz 28850, Spain;

James Finnigan- Email: james.finnigan@prozomix.com, https://orcid.org/0000-0002-8514-784X; **Simon Charnock**- Email: simon.charnock@prozomix.com, https://orcid.org/0000-0003-4437-7419. Prozomix Ltd., Building 4, West End Ind. Estate, Haltwhistle, Northumberland NE49 9HA, United Kingdom;

***Aurelio Hidalgo**- Centro de Biología Molecular Severo Ochoa, Universidad Autónoma de Madrid-Consejo Superior de Investigaciones Científicas (UAM-CSIC), Nicolás Cabrera 1, Madrid 28049, Spain; Department of Molecular Biology, Universidad Autónoma de Madrid, Campus de Cantoblanco, Madrid 28049, Spain; Instituto de Biología Molecular Universidad Autónoma de Madrid, Nicolás Cabrera 1, Madrid 28049, Spain. Email: aurelio.hidalgo@uam.es, https://orcid.org/0000-0001-5740-5584

1. SUPPLEMENTARY METHODS 2
2. SUPPLEMENTARY FIGURES 4
3. SUPPLEMENTARY TABLES 18
4. SUPPLEMENTARY REFERENCES 21

1. Supplementary Methods

Microfluidics Methods. The microfabrication of polydimethylsiloxane (PDMS) chips was carried out as previously described by Mazutis and colleagues[1]. Briefly, Sylgard 184 polydimethylsiloxane (PDMS) elastomer and curing agent were mixed in a 1:10 ratio, degassed and cast onto a microfluidic mold in a silica wafer (Tekniker, Eibar, Spain) with a 2-inlet, 20 micron flow focusing design (Figure S1). After curing overnight at 65 °C overnight, the PDMS slab was peeled from the mold, inlet and outlet ports were punched with a 1 mm biopsy punch (Kai Industries), and the device was thoroughly cleaned with Scotch tape. The PDMS layer was then bonded to a glass slide using oxygen plasma treatment (40 kHz, 100W, 20 seconds) using a Diener Femto plasma etcher. Finally, the assembled device was rendered fluorophyllic by treatment with a 1% (v/v) solution of trichloro(1H,1H,2H,2H-perfluorooctyl)silane (Merck) in HFE 7500 (3M). Water-in-oil droplet emulsions with an average droplet diameter of 20 µm were generated by the convergence of the aqueous phase at 100 µl h^{-1} and 1.5% w/v Pico Surf-1 (Sphere Fluidics) in HFE7500 at 1000 µl h^{-1}. Flow rates were established using a Nemesys Base 120 microfluidic pumps module, controlled by Nemesys software (Cetoni GmbH). Aqueous and oil phases were introduced in the chip with polyethylene tubing of 1.09 mm outer diameter and 0.5 mm inner diameter (Smiths Medical) connected to 100, 1000, 2500, and 5000 µL glass syringes depending on the volume of the experiment. Droplets were collected in an Eppendorf tube.

To generate hydrophylic devices according to Zinchenko and colleagues[2], newly bonded 20 micron flow focusing devices were rendered hydrophilic by successive treatment with 2 mg/mL polydiallyldimethyl ammonium chloride (PDADMAC, Sigma) in 0.5 M NaCl for 10 min, flushing with 150 mM NaCl, 10 min incubation with 2 mg/mL polystyrene sodium sulfonate (PSS, Sigma) in 0.5 M NaCl and flushing with ultrapure water. Then, w/o/w emulsions were generated in two-inlet flow focusing chips flowing a pre-packed w/o emulsion at 40 µL/h and a continuous phase of 1% (v/v) Tween 80 in phosphate-buffered saline (PBS) at 100 µL/h. Flow rates were regulated by a Nemesys Base 120 microfluidic pumps module, controlled by Nemesys software (Cetoni GmbH).

Bacterial Strains and Cultivation. *Escherichia coli* strains were grown in lysogeny broth (LB) medium at 180 rpm, 37 °C for 12 h unless differently specified. If required, the medium was solidified by adding 1.5 % (w/v) of agar. The selection of transformed clones was carried out by growth in the presence of 100 µg/mL ampicillin (Amp) or 30 µg/mL kanamycin (Kan).

Synthesis of 1-(6-(4-methylpiperazin-1-yl)naphthalen-2-yl)ethan-1-one (6). To a solution of 1-methylpiperazine (8 mL, 72 mmol, 7 eq) in dry THF/DMPU 2:1 (v/v) was added dropwise n-BuLi (2.5 M in hexanes, 32 mL, 81 mmol, 8 eq) at 0 °C under Ar. After 15 min, a solution of 1-(6-methoxynaphthalen-2-yl)ethan-1-one (3; 2 g, 10 mmol) in THF was added. The mixture was stirred at room temperature overnight, quenched with cold water, and extracted with DCM. The combined organic layers were dried over $MgSO_4$, filtered, concentrated *in vacuo*, and purified by flash column chromatography to give the piperazinyl substituted ketone **5** (900 mg, 3.3 mmol, 34%).

^{1}H NMR (300 MHz, CDCl3) δ 7.64 (dd, J = 5.2, 3.3 Hz, 3H), 7.39 (dt, J = 8.5, 2.7 Hz, 1H), 7.20 (dd, J = 9.0, 2.4 Hz, 1H), 7.04 (d, J = 2.4 Hz, 1H), 3.35 – 3.20 (m, 4H), 2.89 (s, 1H), 2.61 (t, J = 5.0 Hz, 5H), 2.35 (s, 3H).

^{13}C NMR (75 MHz, CDCl3) δ 173.65, 148.97, 136.26, 128.62, 128.34, 127.12, 125.83, 125.25, 119.52, 110.26, 65.32, 55.00, 49.19, 45.98.

Synthesis of 4-(6-acetylnaphthalen-2-yl)-1,1-dimethylpiperazin-1-ium iodide (4). Compound **4** was prepared by dissolving 1.1 g of ketone **5** (4 mmol, 1 eq) in 30 mL DCM, followed by the addition of 400 µL methyl iodide (6.4 mmol, 1.5 eq). The reaction mixture was stirred overnight and subsequently extracted with water. The solution was lyophilized to give the desired product as a yellow solid in nearly quantitative yield.

^{1}H NMR (300 MHz, methanol-d_4) δ 8.54 (s, 1H), 8.04 (d, J = 9.1 Hz, 1H), 8.01 – 7.83 (m, 2H), 7.53 (d, J = 9.1 Hz, 1H), 7.41 (s, 1H), 3.76 (dd, J = 16.3, 5.5 Hz, 8H), 3.35 (s, 5H), 2.90 (s, 2H), 2.75 (s, 3H). (Figure S3)

^{13}C NMR (75 MHz, methanol-d_4) δ 202.41, 150.55, 132.13, 131.71, 128.34, 125.29, 120.32, 111.15, 62.49, 52.25, 43.85, 36.11, 26.79. (Figure S4)

Synthesis of 4-(6-(1-hydroxyethyl)naphthalen-2-yl)-1,1-dimethylpiperazin-1-ium iodide (*rac*-3). Compound ***rac*-3** was prepared by dissolving 500 mg of ketone **5** (1.22 mmol, 1 eq) in 1 mL of MeOH, followed by the addition of 184 mg $NaBH_4$ (4.87 mmol, 5 eq) under vigorous stirring. After complete conversion the mixture was concentrated and purified by preparative HPLC using water and acetonitrile, containing 0.1% FA, as eluents. The separated fractions were lyophilized to give the desired product as a colorless solid in nearly quantitative yield.

^{1}H NMR (500 MHz, methanol-d_4) δ 7.77 (dd, J = 14.5, 8.7 Hz, 2H), 7.72 (s, 1H), 7.47 (d, J = 8.5 Hz, 1H), 7.35 (dd, J = 9.0, 2.3 Hz, 1H), 7.30 (d, J = 2.3 Hz, 1H), 4.95 (q, J = 6.7 Hz, 1H), 3.65 (dd, J = 18.4, 5.7 Hz, 9H), 3.29 (s, 6H), 1.51 (d, J = 6.5 Hz, 3H). (Figure S5)

^{13}C NMR (126 MHz, methanol-d_4) δ 148.44, 143.21, 135.14, 130.42, 130.13, 128.22, 125.74, 124.55, 120.38, 112.19, 62.83, 52.03, 44.82, 25.48. (Figure S6)

Construction of a soil metagenomic library. Environmental DNA was extracted with the FastDNA Spin Kit for Soil (MP Biomedicals) and digested with Sau3a. DNA fragments between 1 – 8 kbp were isolated and cloned into the BamHI restriction site in plasmid pBlueScript SK (+). Electrocompetent ElectroMAX™ *E. coli* DH10B (ThermoFisher Scientific) aliquots were transformed each with 2 µL of DNA ligation mixture. All transformation reactions were pooled in LB Amp, diluted 1:100, incubated overnight at room temperature, concentrated to 40 ml, aliquoted and stored at -80 ºC in 15 % (v/v) glycerol. To determine the number of unique clones in the library, the 1:100 dilution of the pooled transformations was plated in triplicate on LB Amp supplemented with IPTG and X-gal. To determine the average insert size as well as the probability of finding a full coding sequence within the library, we sequenced the plasmids extracted from cultures inoculated with 25 different white colonies and predicted the encoded ORFs using SnapGene (GSL Biotech), disregarding ORFs encoding proteins smaller than 100 amino acids or truncated proteins.

2. Supplementary Figures

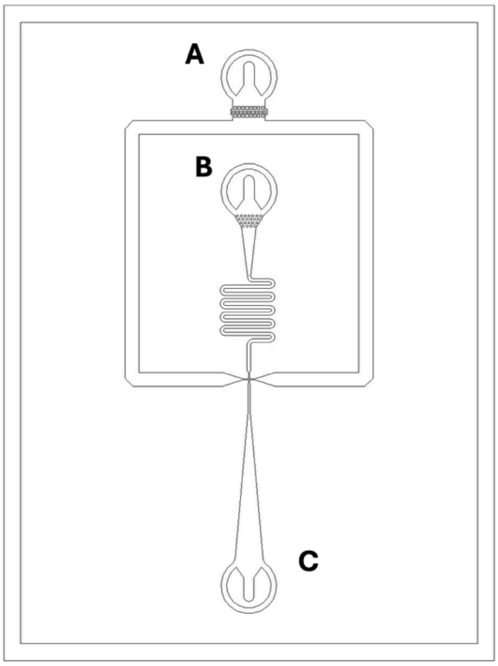

Figure S1. Design of the flow focusing device for the generation of monodisperse water-in-oil (w/o) or water-in-oil-in-water (w/o/w) droplets. To make w/o emulsions, the oil-surfactant mixture was injected through port 1, the aqueous solutions were injected into port 2, and w/o droplets were collected from port 3. To make w/o/w emulsions, the oil-surfactant mixture was injected through port 1, the w/o emulsion was injected into port 2, and w/o/w droplets were collected from port 3. The depth and width of the junction is 20 µm. Design courtesy of the Hollfelder Group (U. of Cambridge)

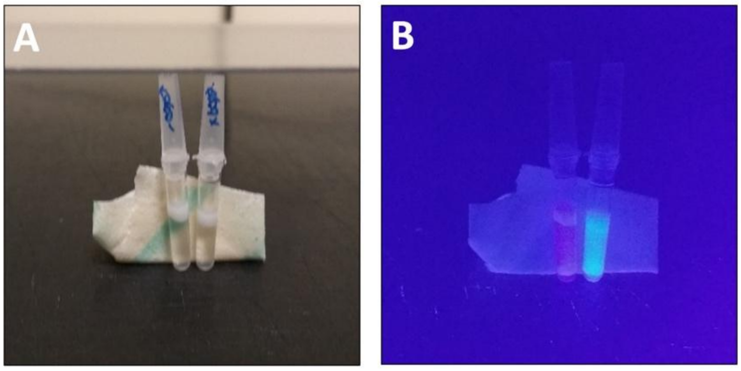

Figure S2. Transfer of the fluorescent ketone 2 in water/oil emulsions. The left tube contains droplets with autoinduction medium and the right tube contains a 1:1 (v/v) mixture of droplets either containing autoinduction medium or autoinduction medium and ketone **2** (A). UV imaging revealed that within 1 minute, the fluorescent ketone **2** had leaked from the droplets containing it (B).

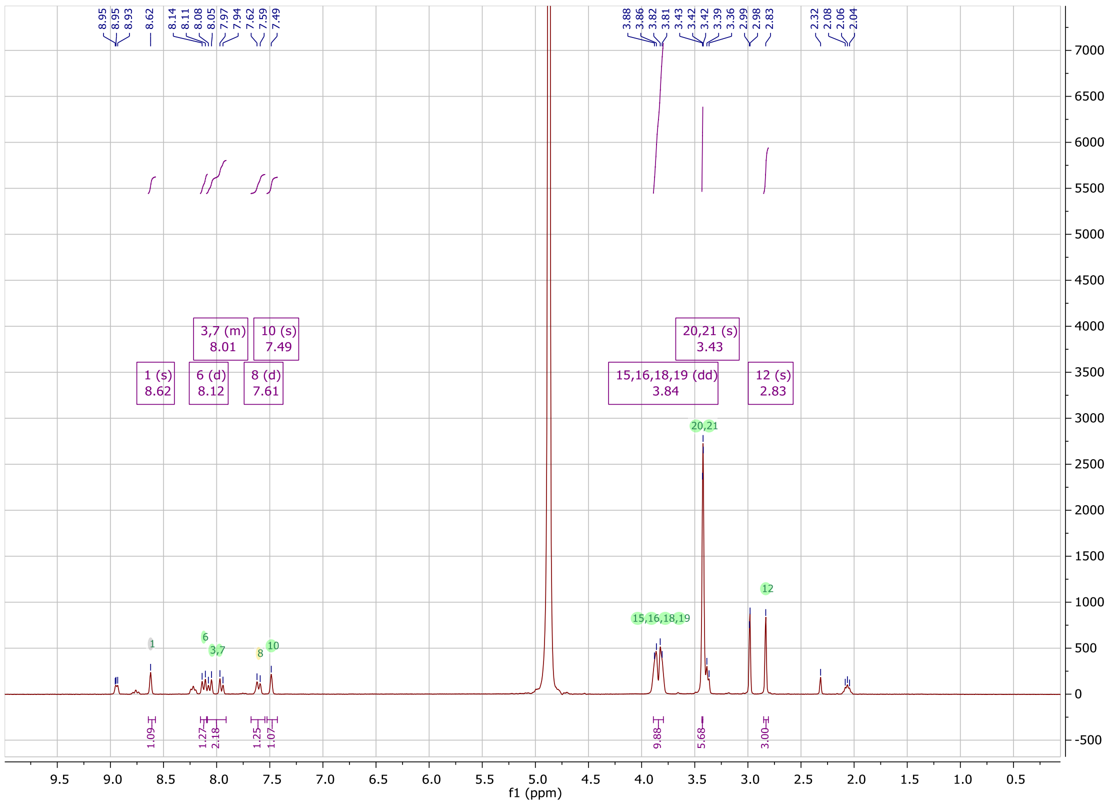

Figure S3. ^{1}H NMR spectrum of ketone **4**

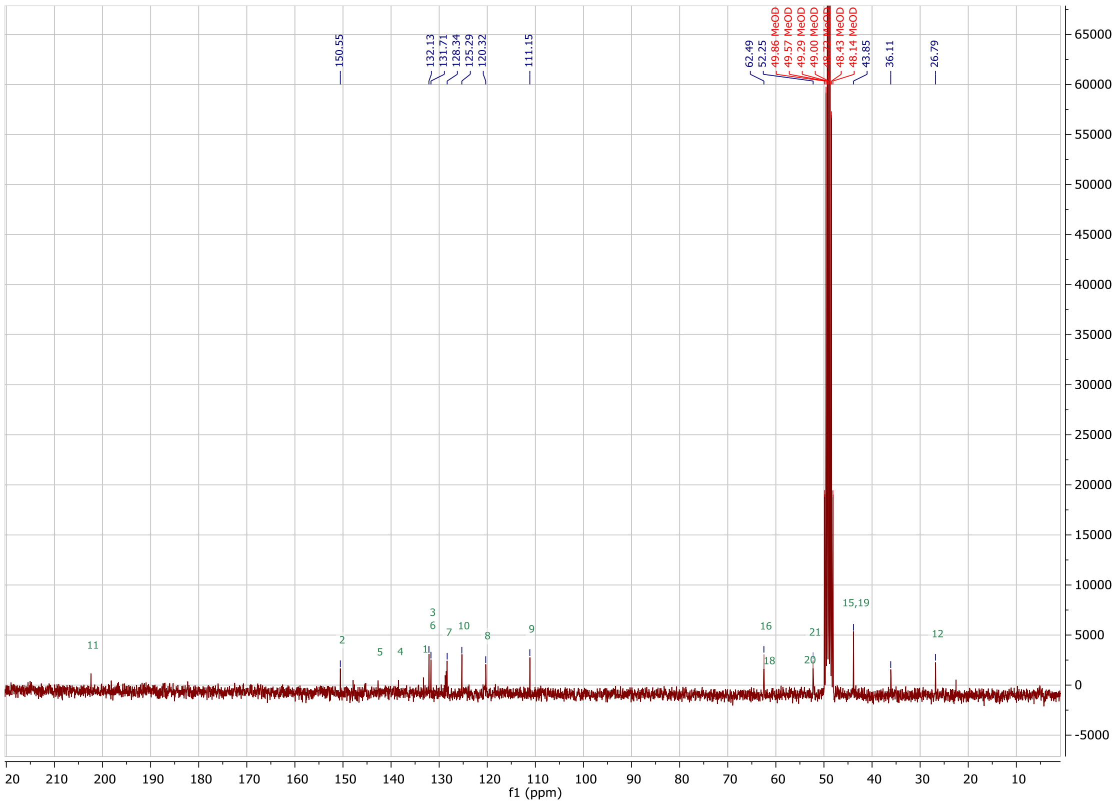

Figure S4. ^{13}C NMR spectrum of ketone **4**

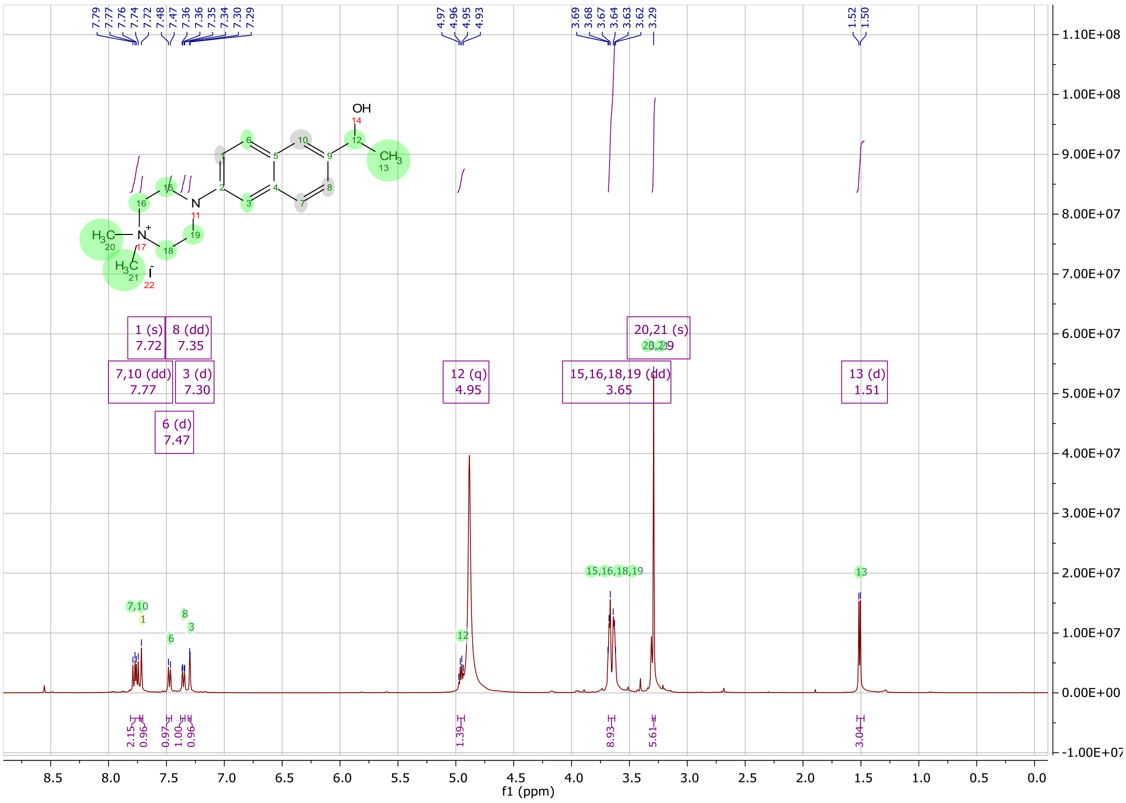

Figure S5. ^{1}H NMR spectrum of secondary alcohol *rac*-**3**

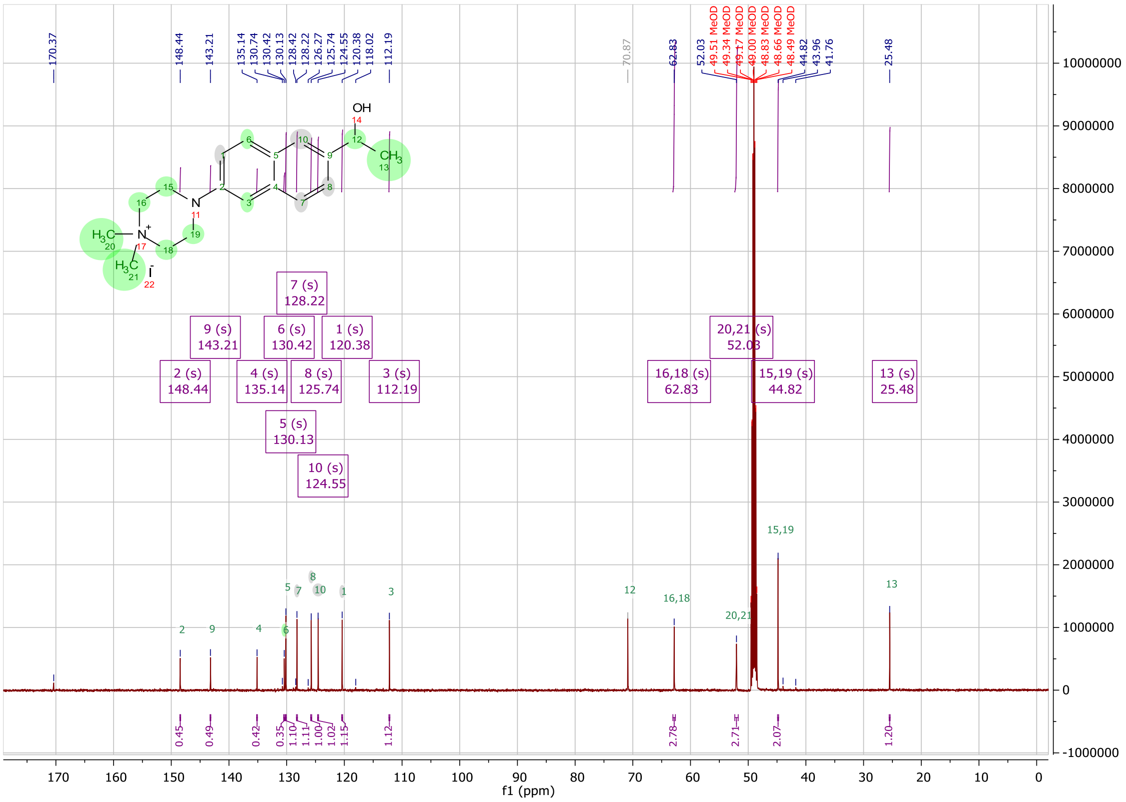

Figure S6. ^{13}C NMR spectrum of secondary alcohol *rac*-**3**

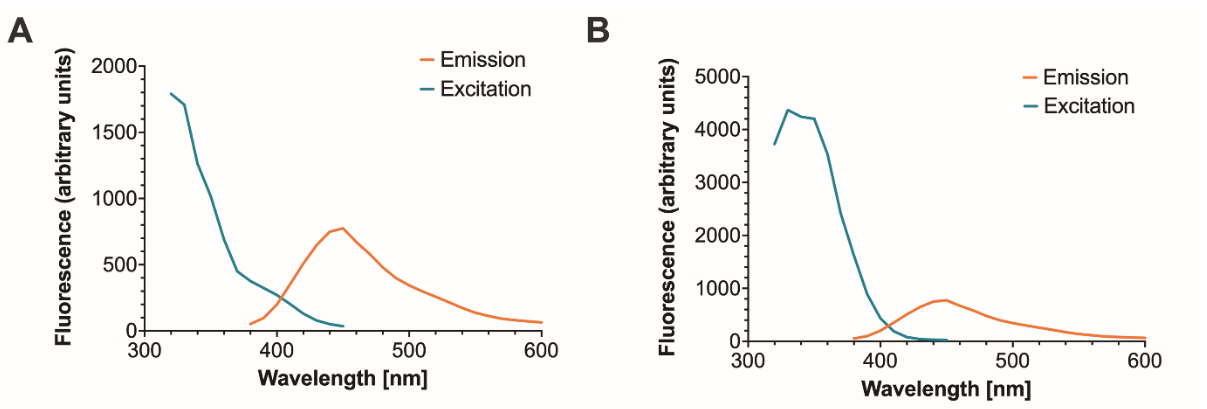

Figure S7. Spectral properties of the original and modified fluorescent products of the KRED assay. Excitation and emission spectra of ketones **2** (A) and **4** (B). All graphics represent the mean of three independent determinations.

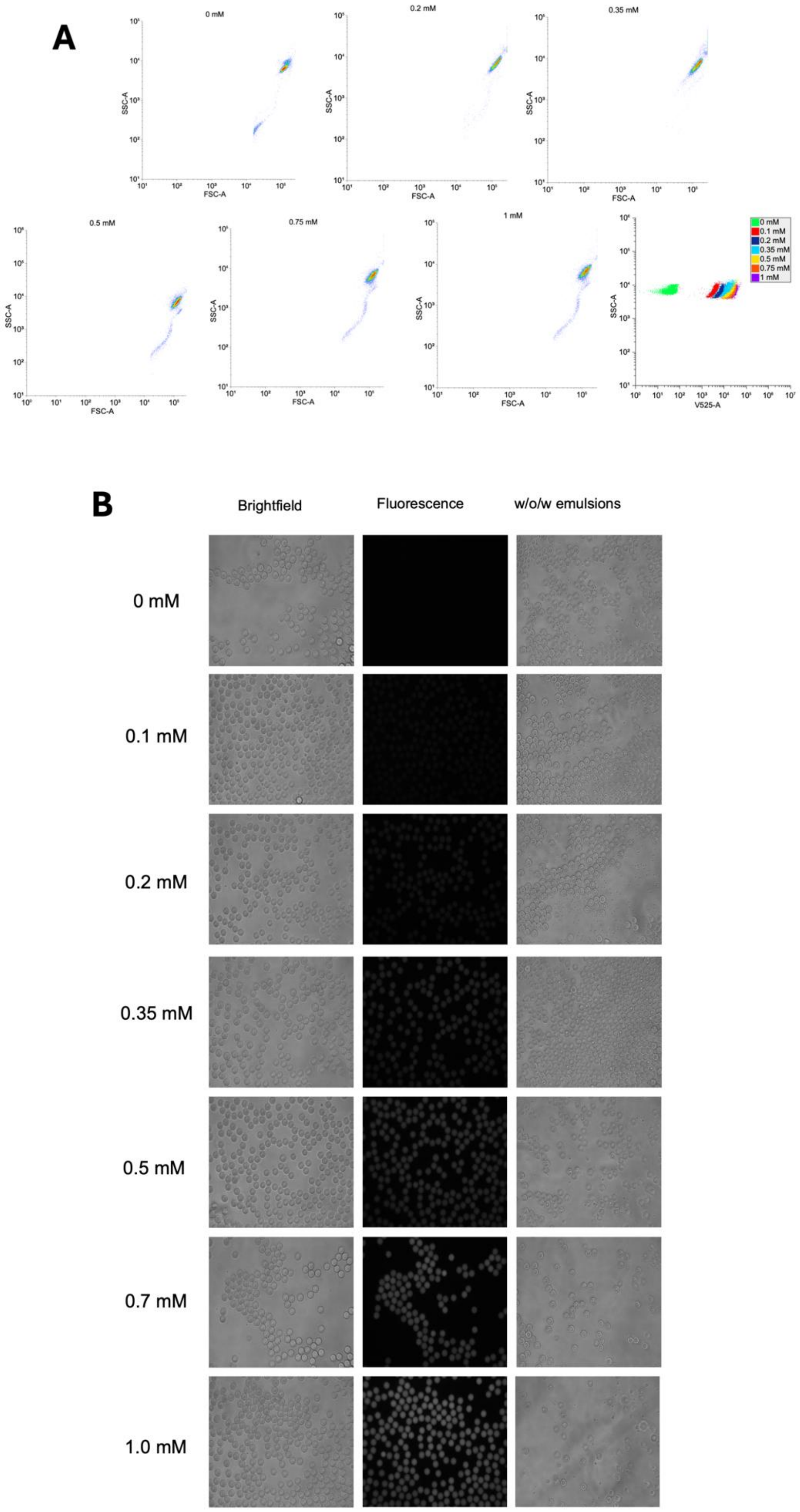
A
0 mM
0.2 mM
0.35 mM
0.5 mM
0.75 mM
1 mM
SSC-A
FSC-A
V525-A
0 mM
0.1 mM
0.2 mM
0.35 mM
0.5 mM
0.75 mM
1 mM
B
Brightfield
Fluorescence
w/o/w emulsions
0 mM
0.1 mM
0.2 mM
0.35 mM
0.5 mM
0.7 mM
1.0 mM

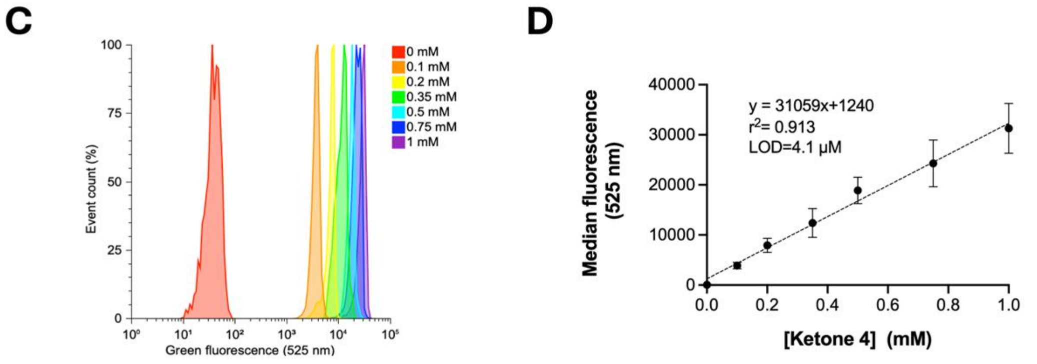

Figure S8. Images and FACS analysis corresponding to the calibration curve of ketone 4. Water-in-oil-in-water droplets were analyzed by FACS and at least 7500 containing several dilutions of ketone 4 were selected for calculation (A). The increase in fluorescence and the quality of the emulsions was verified by microscopy imaging (B). Exposure time: 30 ms. Scale 100 µm. Histogram representation of >7500 water-in-oil-in-water droplets containing several dilutions of ketone **4** analyzed by FACS (C) and their median values plotted against the concentration to establish a calibration curve and determine the limit of detection as LoD= 3.3· σ slope/slope (D).

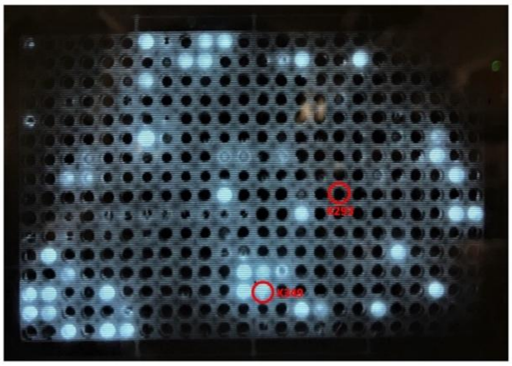

Figure S9. Screening of Prozomix KRED panels 1-4 against alcohol **3**. The image was acquired in a UV-imaging device after 1h of incubation with alcohol **3**. KREDs 349 and 293 were chosen respectively as positive and negative controls for assay development.

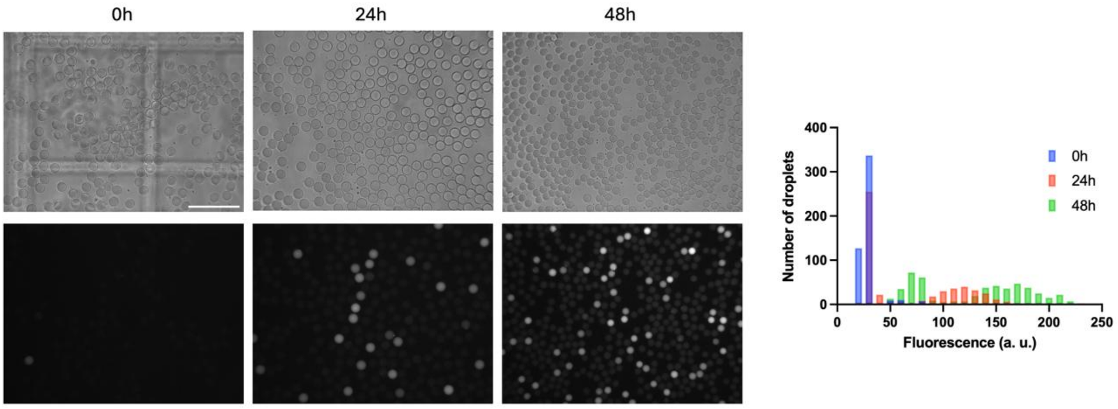

Figure S10. Time course of the conversion of alcohol **3** in water-in-oil-in droplets containing cells expressing K349. The fluorescence of at least 500 droplets was analyzed using Fiji software. Exposure time: 30 ms. Scale 100 µm.

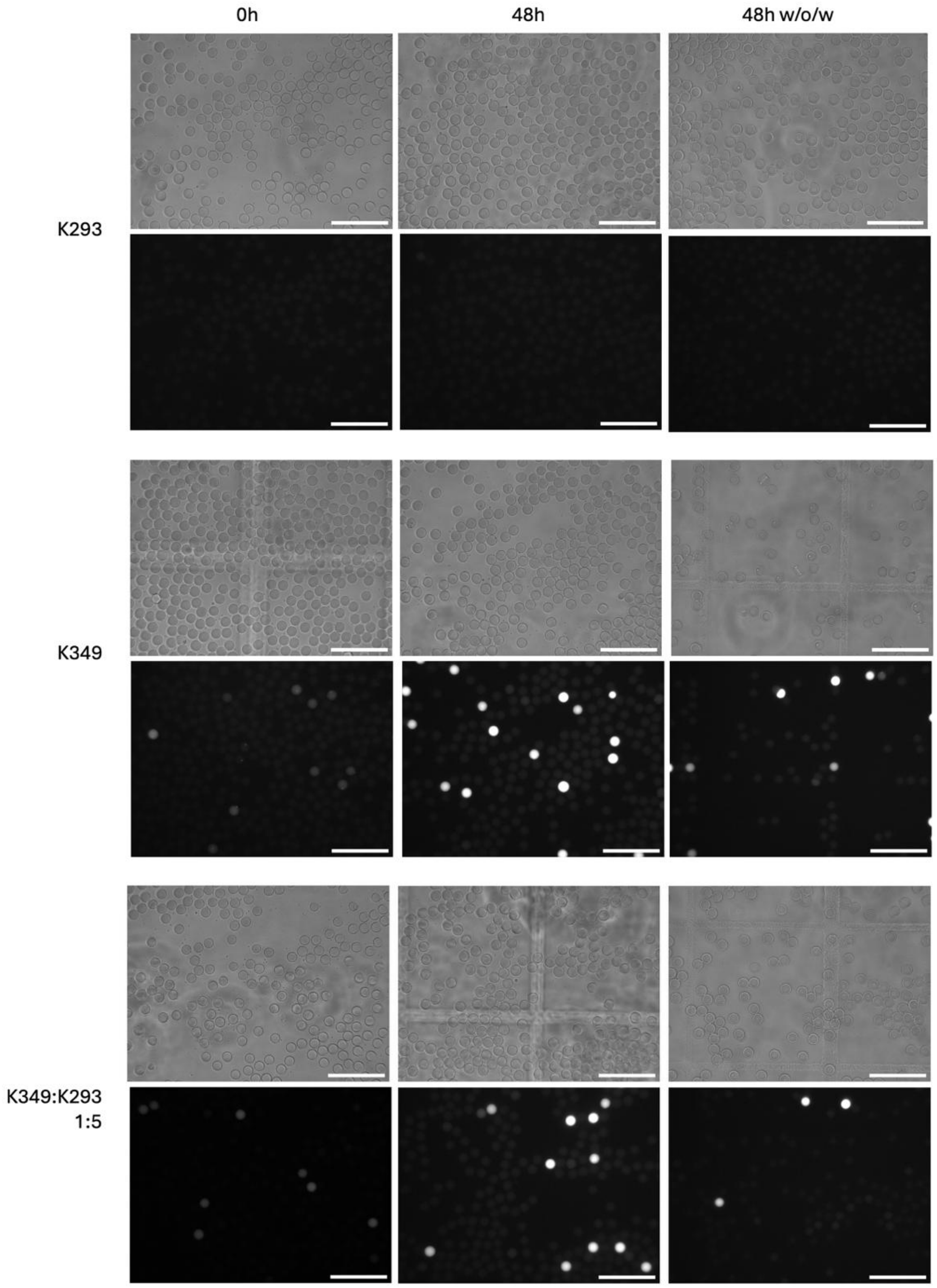

Figure S11. Images corresponding to the emulsions harboring single cells expressing the control KREDs. Water-in-oil-in droplets containing cells expressing K293 or K349 or a mixture of both were imaged at 0 and 48 h of incubation. Water-in-oil-in-water droplets were imaged before FACS sorting for quality check. Exposure time: 30 ms. Scale 100 μm.

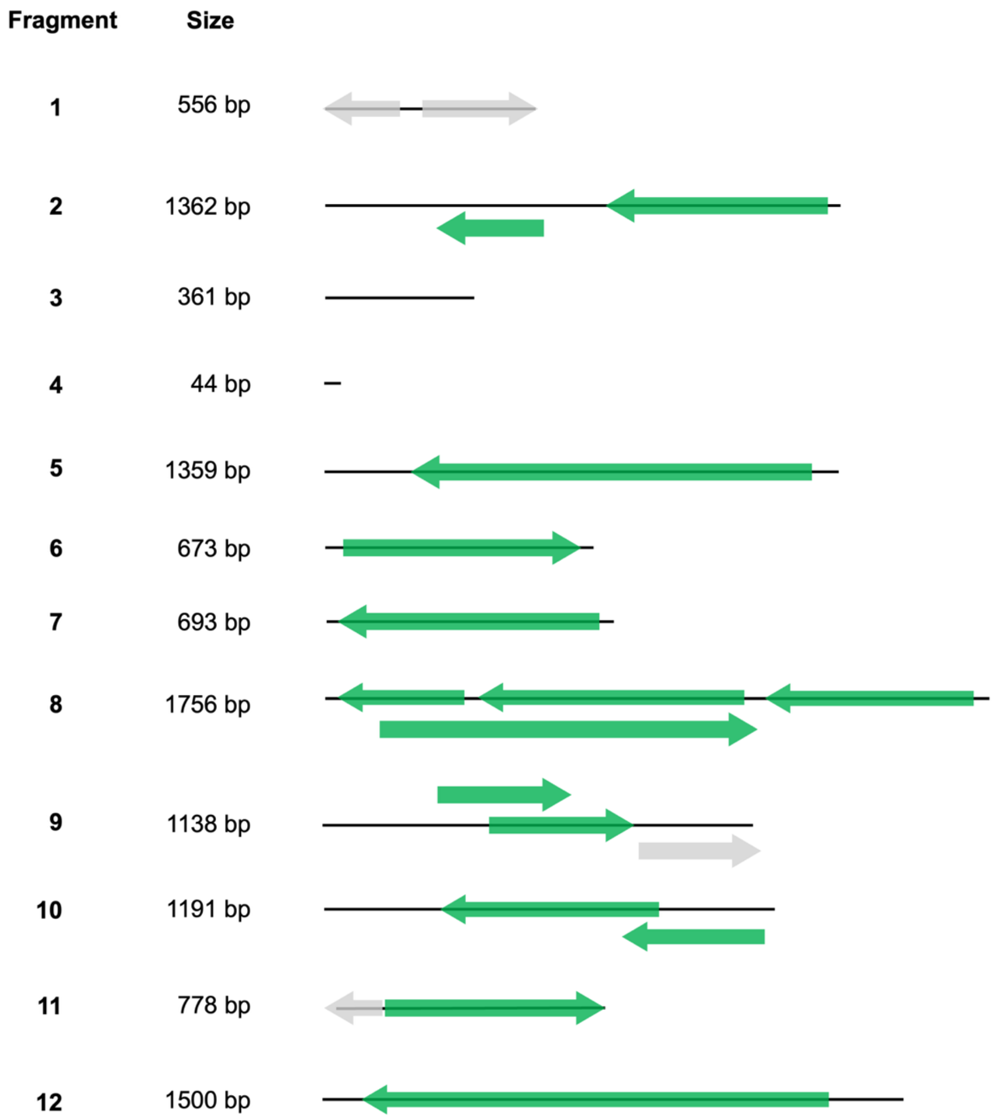
Fragment
Size
1
556 bp
2
1362 bp
3
361 bp
4
44 bp
5
1359 bp
6
673 bp
7
693 bp
8
1756 bp
9
1138 bp
10
1191 bp
11
778 bp
12
1500 bp

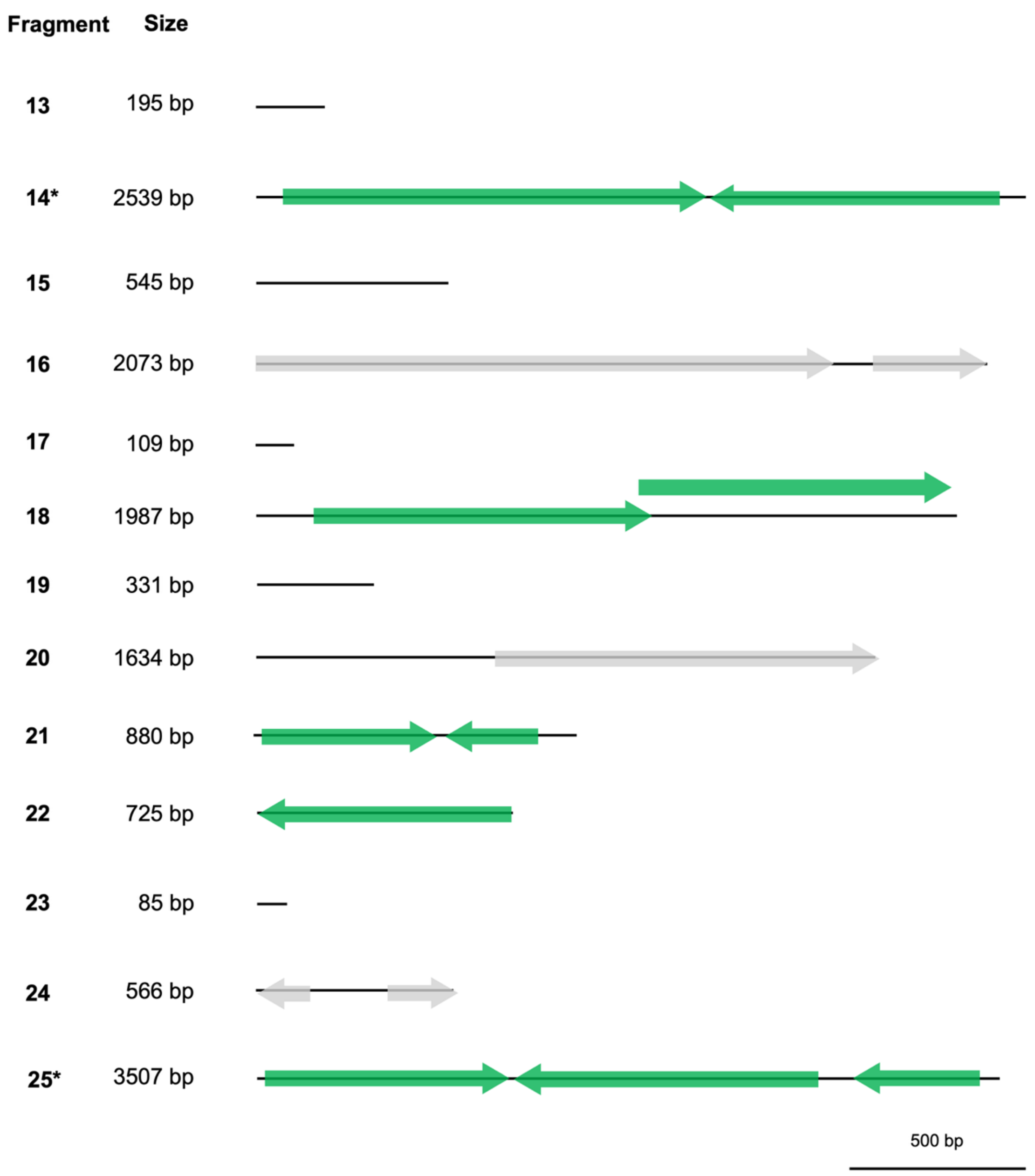

Figure S12. Graphic representation of the chosen metagenomic fragments and their predicted ORFs. 25 randomly picked clones from the library were sequenced and analyzed with SnapGene and BLAST to evaluate the number of whole (in green) or partial ORFs (in grey). All fragments and ORFs are represented to scale except for clones 14 and 25. A 500 bp reference is shown at the bottom right.

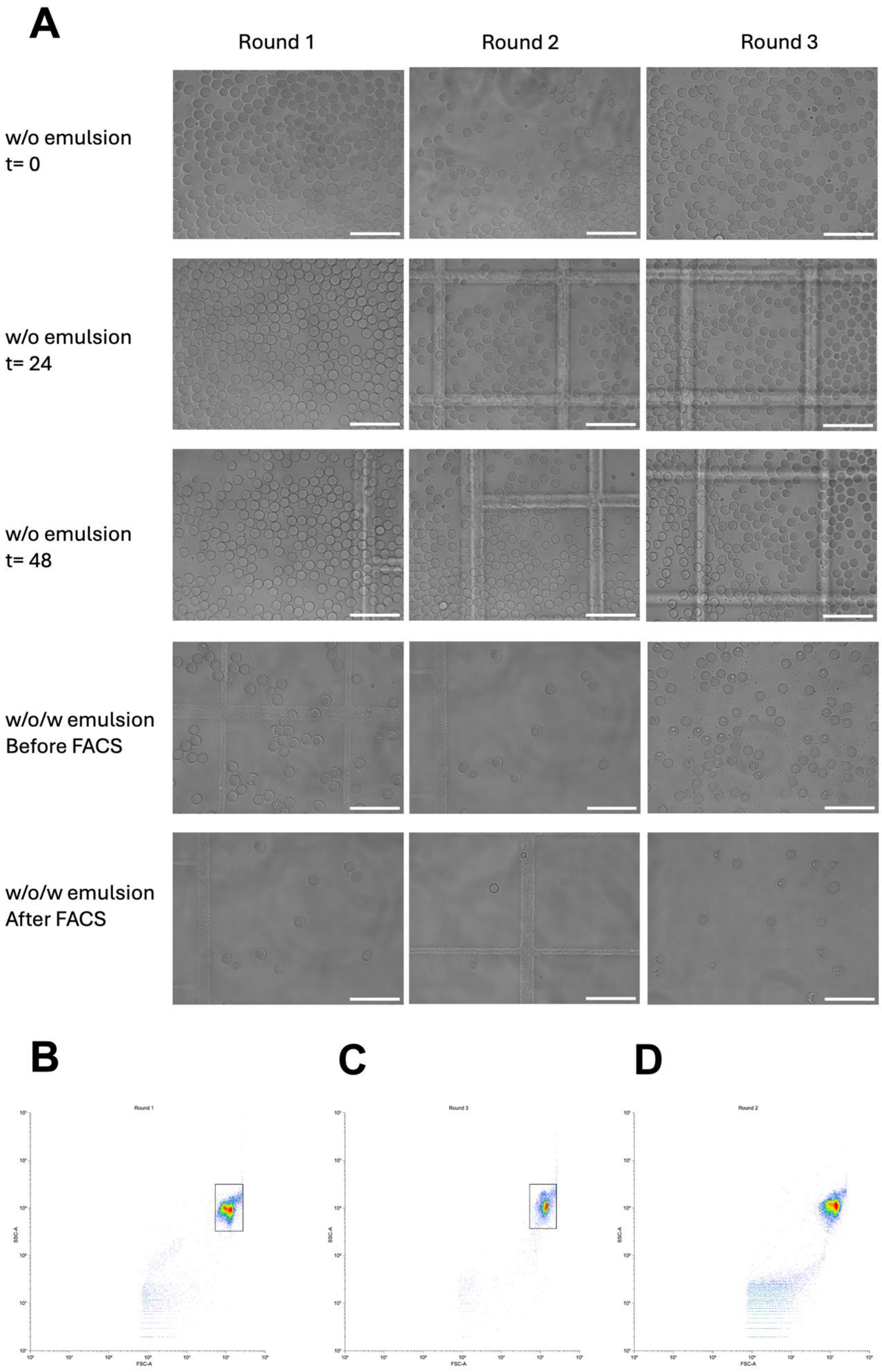
A
Round 1
Round 2
Round 3
w/o emulsion
t= 0
w/o emulsion
t= 24
w/o emulsion
t= 48
w/o/w emulsion
Before FACS
w/o/w emulsion
After FACS
B
C
D
Round 1
Round 3
Round 2
SSC-A
FSC-A

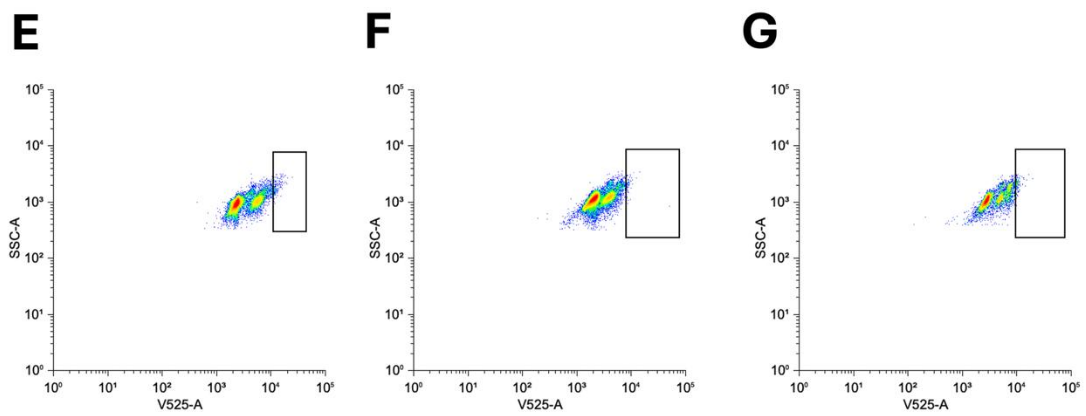

Figure S13. Images and FACS analysis corresponding to the 3 rounds of sorting of the metagenomic library. Water-in-oil-in-water droplets containing the metagenomic library were imaged after 0, 24 and 48 h of incubation and water-in-oil-in-water droplets were imaged before and after FACS sorting to verify integrity and quality of the emulsion. Scale 100 μm. (A) Dot-plots of side scatter (SSC) versus forward scatter are shown for round 1 (B), round 2 (C) and round 3 (D) and green fluorescence at 525 nm of w/o/w emulsions are shown for round 1 (E), round 2 (F) and round 3 (G). The sorting windows shown in panels E-G were established to recover 0.85%, 0.24% and 1.69 % of the analyzed droplets with the highest fluorescence in each round, respectively

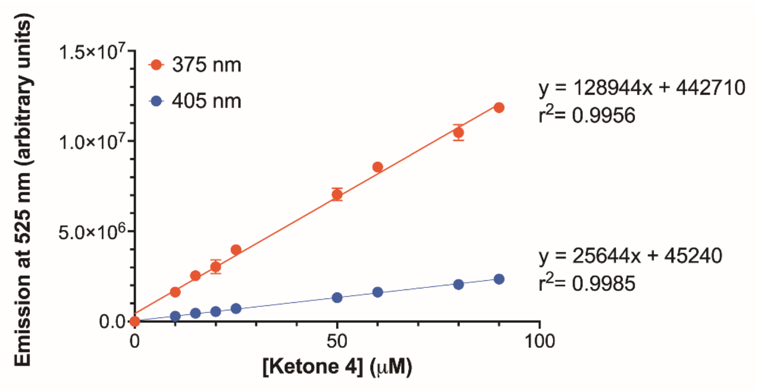

Figure S14. Calibration curve of ketone **4**. The calibration curve of ketone **4** at 525nm using 375 or 405 nm as excitation wavelengths commonly found in FACS instruments. Points represent the mean of three independent determinations. Error bars represent the standard deviation.

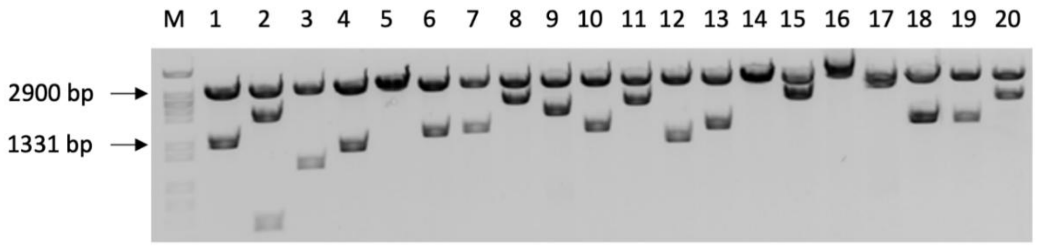

Figure S15. Restriction analysis of plasmids from FACS-positive metagenomic library clones. The plasmids from 20 randomly FACS-positive clones of the metagenomic library (lanes 1-20) were purified and digested with the restriction enzymes XbaI-HindIII. The reactions were analyzed by DNA electrophoresis in a 1% agarose gel. M: Φ (HindIII) DNA ladder, where DNA fragments of 2900 and 1331 bp have been indicated with arrows for easier interpretation.

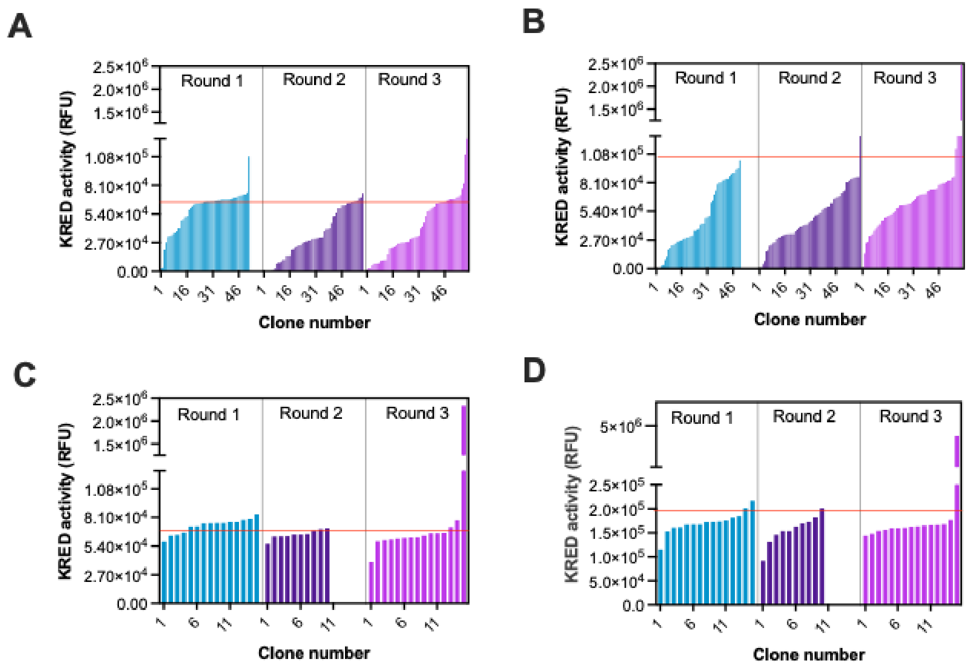

Figure S16. Blank-corrected enzymatic activity assays of *E. coli* clones from the positive population of the sorted droplets from the three rounds of screening. A minimum of 50 colonies from each round were grown and assayed with alcohol **3** and NAD^+ (A) or $NADP^+$ (B) in a microtiter plate. Confirmation assay with NAD^+ (C) or $NADP^+$ (D) in *E. coli* DH5α transformed with the plasmids recovered from the positive hits. Red lines indicate the stringent arbitrary threshold ($\bar{x}$blank+5 σ blank) used for considering positive KRED activity. AFU: arbitrary fluorescence units

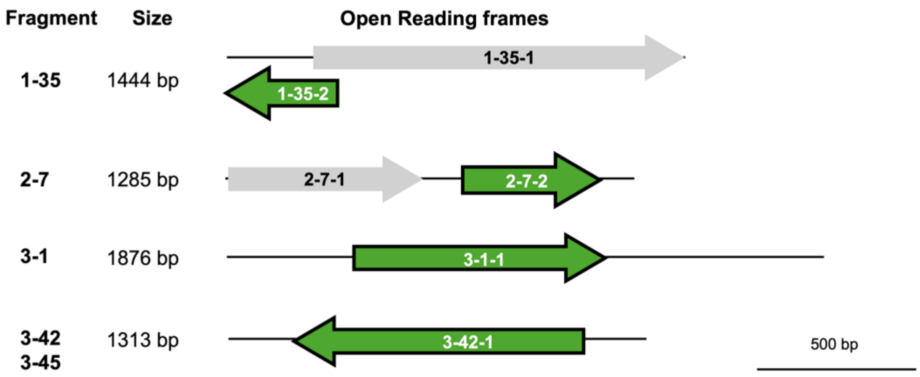

Figure S17. Graphic representation of the chosen metagenomic fragments and their predicted ORFs. All putative KRED-encoding ORFs for cloning are colored in green). All fragments and ORFs are represented to scale. A 500 bp reference is shown at the bottom right.

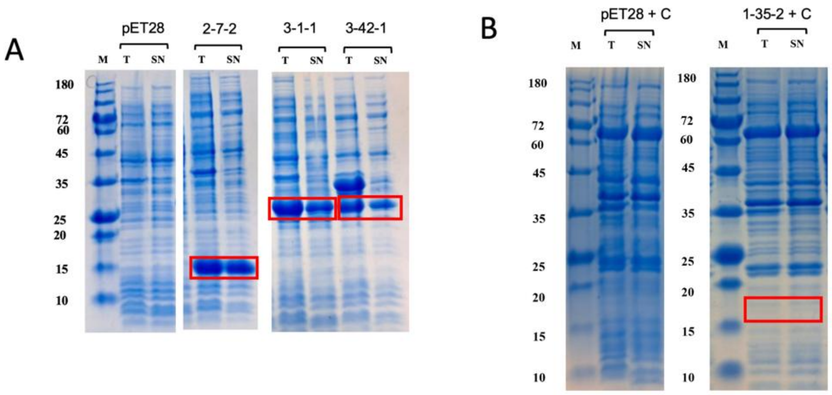

Figure S18. SDS-PAGE analysis of the expression of the discovered KREDs. Proteins were expressed in the absence (A) or in the presence (B) of co-expressed DnaK, DnaJ, and GrpE chaperones. C: co-expressed chaperones. M: molecular weight protein ladder. T and SN: total and supernatant fractions, respectively. The numbers 1-35-2, 2-7-2 3-1-1 and 3-42-1 refer to the expressed ORFs inside the pET28 vector. Expected bands are indicated with a red rectangle.

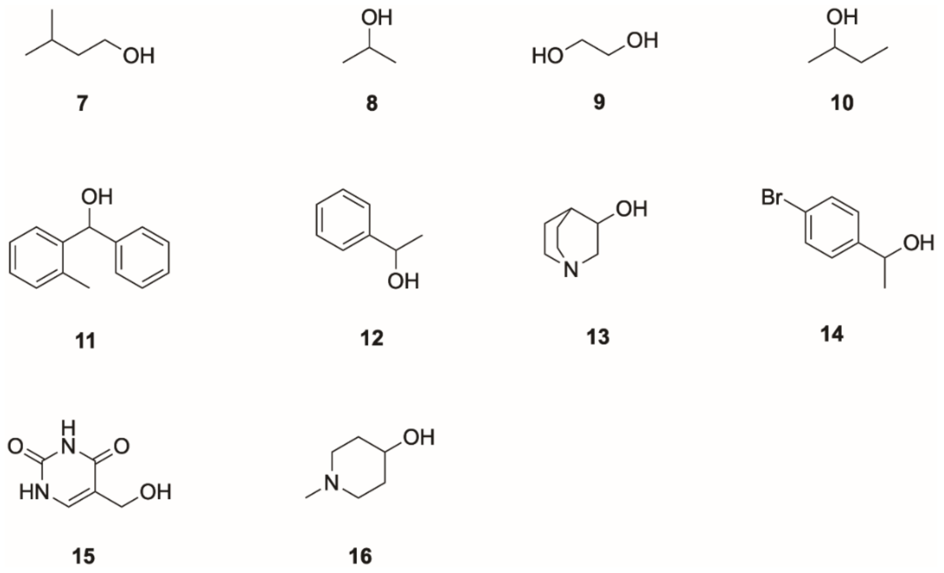

Figure S19. A panel of structurally diverse alcohols of relevance in biocatalysis for the characterization of the discovered proteins with KRED activity. A set of 10 alcohols with structural diversity (aliphatic, aromatic, primary, secondary, linear, and cyclic) were chosen to probe the substrate specificity of the proteins with KRED activity that were recombinantly expressed and purified.

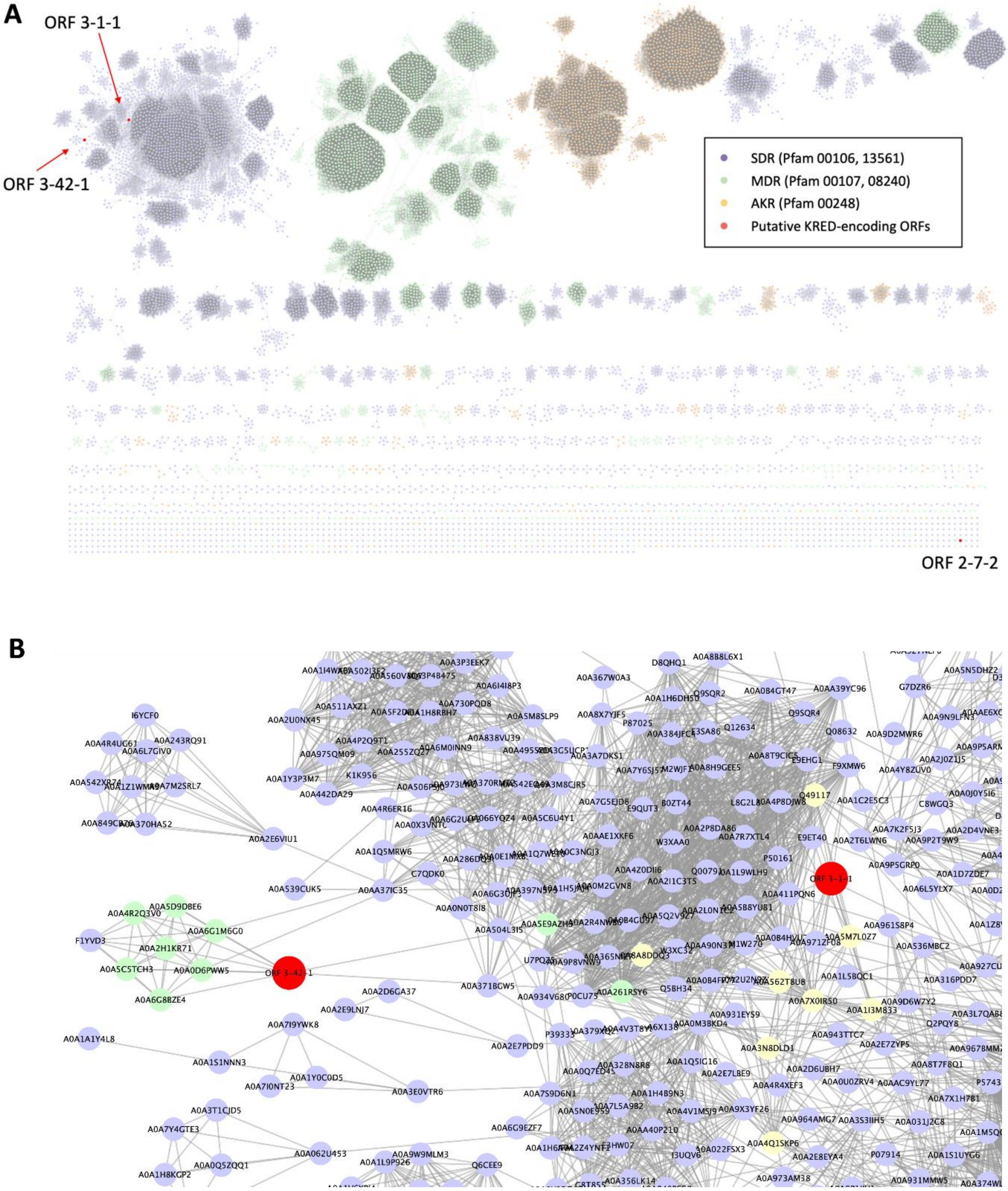

Figure S20. Sequence similarity network (SSN) of KRED families. SSN of >10000 bacterial representatives of the 3 main KRED families (Short- and medium-chain dehydrogenase/reductase [SDR and MDR], and aldo-keto reductases [AKR]) and our putative KRED-encoding ORFs (in red) (A). Connectivity of hits 3-1-1 and 3-42-1 (connected nodes in yellow and green, respectively) (B). Node labels are Uniprot entries. Sequences with 100% identity have been grouped into a single node. Edges signify sequence identity >40%.

3. Supplementary Tables

Table S1. Oligonucleotides used for sequencing.

**Name**	**Sequence (5’ → 3’)**
T7_prom_fw	TAATACGACTCACTATAGGG
T7_term_rv	GCTAGTTATTGCTCAGCGG
Plac_fw	CCGGCTCGTATGTTGTGTGG
M13_fw	GTAAAACGACGGCCAGT
M13_rv	CAGGAAACAGCTATGAC

Table S2. Plasmids used in this work.

**Plasmid**	**Features**	**Purpose**
pET28b(+) Novagen	KanR, T7 promoter	Vector for gene expression from RNA polymerase under the promoter of T7 phage in *E. coli*. Includes a His-tag (6 histidines) at the N-or C-terminal site.
pBluescript SK(+) Agilent	standard cloning vector (phagemid excised from lambda ZAP); AmpR, *lac* promoter	Enables white/blue screening of clones with the lacZα system. Used for cloning of recovered DNA from metagenomic screenings and confirmation with activity assays in solid medium.

Table S3. *Escherichia coli* strains used for this study.

**Strain**	**Purpose**	**Genotype**
E. coli DH5α	Molecular cloning [3]	supE44, ΔlacU169, j80 lacZΔM15, hsdR17, recA, endA1, gyrA96, thi-1, relA1
E. coli BL21(DE3)	Protein expression [4]	hsdS, gal, ind1, Sam7, nin5, lacUV5-T7 gene1
E. coli ElectroMAX™ DH10B	Metagenomic library construction	(F-mcrA Δ(mrr-hsdRMS-mcrBC) Φ80lacZΔM15 ΔlacX74 recA1 endA1 araD139Δ(ara, leu)7697 galU galK λ-rpsL nupG)

Table S4. Culture media used in this work.

**Medium**	**Composition**
Lactose autoinduction medium (ZY medium)	Bacto-triptone 10g/L, yeast extract 5g/L, glycerol 0.5 g/L, glucose 0.5 g/L, lactose 2g/L, (NH4)2SO4 3.3 g/L, KH2PO4 6.8 g/L, Na2HPO4 7.1 g/L, MgSO4 0.12g/L *E. coli* protein expression[5].
LB medium	Bacto-triptone 10 g/L, yeast extract 5 g/L, NaCl 5 g/L, pH 7.0. *E. coli* growth medium.
SOC	Bacto-triptone 20 g/L, yeast extract 5 g/L, NaCl 0.58 g/L, KCl 0.18 g/L, MgCl2 10 mM, MgSO4 0 mM, glucose 2 % (w/v). Transformation of *E. coli* cells[3].

Table S5. Characteristics of the predicted ORFs[a] included in the metagenomic hits. Selected ORFs[a] for cloning are indicated by a shaded row.

ORF	Sequence	BLASTP	Interpro			Phyre2	AlphaFold
			Family membership	Domain	Homologous superfamily		
1-35-1	MTIVKCPKCRKRYDPGSKNQGDSPDHSRKVVCPSCGQWARLPQGDAIKTPNVPPKMVKALMSQARLLDDDDDPSPPTVAPRPDNDDDDEGGAYGFLAEETSAAPAPTKPEKKPKEKKTPVVHRKIKKKTVQFADQWSKIHFALWLFFAGICLWGFCWLLRLVMVMQGLLTAALYSPLQLSVMNSDGQPNLAAFSIGLIVGMENMDLGMAFYITEQSLFLAAGGCFLGGYCVCLGLPNVYGIRGQAITLIVLGNINLVVGLLLRLLPAVGVIGYAMIPLLAPEIAFNVCNLERAIPLTVFWCTSPFWEFLATIILQALFFAEPILFCVLLRSTAMAMKDDHWLEPRAQMLLRIGFGQVFMLLSYYLLSVTGTSEVLVWTLLVIYFVWRCF	Hypothetical protein [Planctomycetota bacterium] (coverage 100%, identity 61.8%)	n.f.	n.f.	n.f.	n.f.	Znf/thioredoxin_put domain containing protein (identity 29.7%)
1-35-2	MVRRIALVLAAGIVALAALGTLDNGHARLQPRTGSDIRQCNSSQLEPRLQRSEGRRTATISHGSPRRLRGGRVCQRRHRQTPRHCLSQHPVIPRRHNERFQHNSATPHKLCAKHLCK	n.f.	n.f.	n.f.	n.f.	Oxidoreductase (conf. 7.9% / identity 44%)	Hypothetical protein UA=A0A820B8A1
2-7-1	DLSPRAYPPLAKALALGEVDTFLQRRSDVRAAERRLASATANEGIAAADLYPRITVTGFLGFLAGRGNLLFESDSRAWAVTPALSWAAFDLGSARARLRGAEAGTREALALYEQTILRALEETENAFVNYREQQQRLVKLTDQARESARASAIARARYREGVSDFLALLDAERTQLQAENAVAQAEAQVFTSVVGVYKTLGGI*	efflux transporter outer membrane subunit [Thermoanaerobaculia bacterium] (coverage 99%,identity 83%)	n.f.	n.f.	n.f.	n.f.	n.d.

ORF	Sequence	BLASTP	Interpro			Phyre2	AlphaFold
			**Family membership**	**Domain**	**Homologous superfamily**		
2-7-2	MDVSEMTKRLRNKIALIASTAGTG<br>VAAARRFAAEGAEVIAISHDADAL<br>AIARRELDGVARVVQLDPADERSV<br>THFFAVLGRKHGRLDVLFLNAGFG<br>ALNHAIPLLSEGAAVIVDGSDAAPI<br>MFCASRDSSTILGAELAVGGKA*	SDR family oxidoreductase (coverage 56%, identity 37.3%)	SDR IPR002347 PF00106	n.d.	NAD(P) binding IPR036291	oxidoreductase	n.d.
3-1-1	MFLCEAFRRRRVMSKKLSGKVAV<br>VTGASKGIGAEIARQLAAAGAAVV<br>VNYSSSKEGADQVVADIVGREGK<br>ALAVRANLASHEDVQRLFAEAKQ<br>AFGRLDVLVNNAGIYEFAPLDALT<br>AEHFHKQFDLNVLGLLWATQEAV<br>KQFGPEGGSIINISSVAATVAPPTAS<br>VYSATKAAVNAVTRSLGQELGAR<br>KIRVNSINPGMVETEGFHASGVFGS<br>NFHTATEAQTPLGRIGQPQDIAPLA<br>VFLASSDSAWITGESFYVSGGLR*	Glucose 1-dehydrogenase [Planctomycetota bacterium] (coverage 95%, identity 78.8%)	Short-chain dehydrogenase/reductase SDR	adh_short_C2 (PF13561)	NAD(P)-binding Rossmann-fold domains	Oxidoreductase (glucose dehydrogenase)	Glucose 1-DH
3-42-1 / 3-45-1	>ORFS 3-42-1 3-45-1<br>MTTNDFTGANPSRDELSGGGMPR<br>RQALMGLGALLFAAAGARTAAAQ<br>TARTTTAKDLAGKVAIVTGARNNL<br>GRAFAIALARNGANIVVHYHRAET<br>QAEAEETASLVRAEGVKAVLVQG<br>DLSIVANIRKMYELAMNEFGRVDI<br>VVNNAGYIKKKPFVEITEEEFDRC<br>VGINTKGLYFSMQEAAKRIADNGR<br>IINIGTSLLGATTGMYSAYAGTKAP<br>VEAFTRALAKEIGKRGITVNVVAP<br>GAVDTPFFHSQETPETVEYVKKGH<br>VSGRLATVDDIVGTIVFLASPASQ<br>WLSAQTIFVNNAYLAR	SDR family oxidoreductase [Myxococcales bacterium] (coverage 82%, identity 60.2%)	Short-chain dehydrogenase/reductase SDR (IPR002347)	n.f.	NADP-binding IPR036291 SSF51735	oxidoreductase	Short chain dehydrogenase

[a] ORF: open reading frame; [b] n.f.: not found; [c] n.d.: not determined

4. SUPPLEMENTARY REFERENCES

(1) Zinchenko, A.; Devenish, S. R. A.; Kintses, B.; Colin, P.-Y.; Fischlechner, M.; Hollfelder, F. One in a Million: Flow Cytometric Sorting of Single Cell-Lysate Assays in Monodisperse Picolitre Double Emulsion Droplets for Directed Evolution. *Anal. Chem.* **2014**, *86* (5), 2526–2533. https://doi.org/10.1021/ac403585p.

(2) Goldberg, K.; Schroer, K.; Lütz, S.; Liese, A. Biocatalytic Ketone Reduction—a Powerful Tool for the Production of Chiral Alcohols—Part II: Whole-Cell Reductions. *Appl Microbiol Biotechnol* **2007**, *76* (2), 249–255. https://doi.org/10.1007/s00253-007-1005-x.

(3) Hanahan, D. Studies on Transformation of Escherichia Coli with Plasmids. *Journal of Molecular Biology* **1983**, *166* (4), 557–580. https://doi.org/10.1016/S0022-2836(83)80284-8.

(4) Rosenberg, A. H.; Lade, B. N.; Dao-shan, C.; Lin, S.-W.; Dunn, J. J.; Studier, F. W. Vectors for Selective Expression of Cloned DNAs by T7 RNA Polymerase. *Gene* **1987**, *56* (1), 125–135. https://doi.org/10.1016/0378-1119(87)90165-X.

(5) Studier, F. W. Protein Production by Auto-Induction in High-Density Shaking Cultures. *Protein Expression and Purification* **2005**, *41* (1), 207–234. https://doi.org/10.1016/j.pep.2005.01.016.
